# Evidence that molecular changes in cells occur before morphological alterations during the progression of breast ductal carcinoma

**DOI:** 10.1186/bcr2157

**Published:** 2008-10-17

**Authors:** Nadia P Castro, Cynthia ABT Osório, César Torres, Elen P Bastos, Mário Mourão-Neto, Fernando A Soares, Helena P Brentani, Dirce M Carraro

**Affiliations:** 1 Laboratório de Genômica e Biologia Molecular, Centro de Pesquisa Hospital do Câncer A C Camargo, CEP: 01509-900, São Paulo, SP, Brazil; 2 Departamento de Anatomia Patológica, Centro de Pesquisa Hospital do Câncer A C Camargo, CEP: 01509-900, São Paulo, SP, Brazil; 3 Laboratório de Bioinformática, Centro de Pesquisa Hospital do Câncer A C Camargo, CEP: 01509-900, São Paulo, SP, Brazil; 4 Departamento de Mastologia, Hospital do Câncer A C Camargo, CEP: 01509-900, São Paulo, SP, Brazil

## Abstract

**Introduction:**

Ductal carcinoma *in situ *(DCIS) of the breast includes a heterogeneous group of preinvasive tumors with uncertain evolution. Definition of the molecular factors necessary for progression to invasive disease is crucial to determining which lesions are likely to become invasive. To obtain insight into the molecular basis of DCIS, we compared the gene expression pattern of cells from the following samples: non-neoplastic, pure DCIS, *in situ *component of lesions with co-existing invasive ductal carcinoma, and invasive ductal carcinoma.

**Methods:**

Forty-one samples were evaluated: four non-neoplastic, five pure DCIS, 22 *in situ *component of lesions with co-existing invasive ductal carcinoma, and 10 invasive ductal carcinoma. Pure cell populations were isolated using laser microdissection. Total RNA was purified, DNase treated, and amplified using the T7-based method. Microarray analysis was conducted using a customized cDNA platform. The concept of molecular divergence was applied to classify the sample groups using analysis of variance followed by Tukey's test.

**Results:**

Among the tumor sample groups, cells from pure DCIS exhibited the most divergent molecular profile, consequently identifying cells from *in situ *component of lesions with co-existing invasive ductal carcinoma as very similar to cells from invasive lesions. Additionally, we identified 147 genes that were differentially expressed between pure DCIS and *in situ *component of lesions with co-existing invasive ductal carcinoma, which can discriminate samples representative of *in situ *component of lesions with co-existing invasive ductal carcinoma from 60% of pure DCIS samples. A gene subset was evaluated using quantitative RT-PCR, which confirmed differential expression for 62.5% and 60.0% of them using initial and partial independent sample groups, respectively. Among these genes, *LOX *and *SULF-1 *exhibited features that identify them as potential participants in the malignant process of DCIS.

**Conclusions:**

We identified new genes that are potentially involved in the malignant transformation of DCIS, and our findings strongly suggest that cells from the *in situ *component of lesions with co-existing invasive ductal carcinoma exhibit molecular alterations that enable them to invade the surrounding tissue before morphological changes in the lesion become apparent.

## Introduction

Ductal carcinoma *in situ *(DCIS) of the breast is characterized by a proliferation of malignant-appearing epithelial cells of the ducts but without detachment of the basement membrane or evidence of invasion [1]. This disease lies within a spectrum of preinvasive lesions with a vast range of malignant potential. DCIS can progress rapidly to invasive cancer or it may change very slowly [2]. Therefore, an ability to identify which DCIS lesions are likely to progress to invasive carcinoma and over what time interval would greatly enhance treatment selection and outcome in breast cancer patients.

The current view of the malignant process is that cancer cells acquire malignant potential by accumulating alterations that permit them to overcome the strict rules of normal cell growth regulation imposed by their environment [3]. Breast cancer is a multistep process that manifests through a series of pathological stages, namely atypical ductal hyperplasia, DCIS and invasive ductal carcinoma (IDC), the latter being potentially lethal if subsequent development of distant metastasis occurs [4]. Molecular and pathological evidence suggests that DCIS can be precursor to invasive disease (although this is not without exception) [5-11]. However, it is not clear which cell populations progress to invasive disease and what molecular properties give them the capacity to spread to surrounding tissue.

Despite much research effort, the molecular basis of breast cancer tumorigenesis and progression [9,12-17] has not completely been elucidated. Two major approaches have been used to address these issues: oligo/cDNA microarrays and laser microdissection. Microarrays allow researchers to examine the expression of several genes simultaneously, identifying gene sets that discriminate groups of cancer samples with common clinical or pathological characteristics and risk for progression to IDC. Laser microdissection is crucial in permitting the molecular analysis of defined, homogenous cell types from a specific solid tissue. Both methodologies have been used to discover novel prognostic markers and to predict disease outcomes [9,16,18,19].

The pathological classification of DCIS does not accurately predict invasive disease. In the present study we compared the gene expression profiles of cells captured from *in situ *component lesions, pure DCIS, and *in situ *component of DCIS with co-existing IDC (DCIS-IDC), with the goal being to find molecular makers that can predict risk for invasive disease. We also examined epithelial cells of initial (non-neoplastic epithelial cells) and later stages (IDC cells) of ductal carcinoma progression.

The molecular characteristics of cells from the *in situ *component of DCIS-IDC are more similar to cells from IDC than to those from pure DCIS (the latter being morphologically identical), which strongly suggests that their molecular reprogramming precedes morphological alteration in the lesion. Moreover, we identified several candidate genes, including *LOX *[GenBank: NM_002317] and *SULF-1 *[GenBank: NM_001128206], which are putatively involved in the acquisition of the capacity to invade adjacent tissues of DCIS. These genes may serve as molecular markers that can identify those DCIS lesions that may become invasive.

## Materials and methods

### Samples

Fresh-frozen human breast tumor samples were retrieved from the Tumor Tissue Biobank of the Medical and Research Center – Hospital A C Camargo, São Paulo. Sections 5 μm thick from the fresh-frozen tumor blocks were cut onto glass slides, stained with hematoxylin and eosin, and reviewed by a pathologist. The hematoxylin and eosin stained sections were used to evaluate and select appropriate tumor areas corresponding to each histological component. The histological grade of the DCIS was assigned in accordance with the World Health Organization scale [20], and IDC was classified in accordance with the Scarff-Bloom-Richardson grading scheme [21,22]. Forty-one samples were evaluated, consisting of four non-neoplastic breast samples, five pure DCIS (stage 0) samples, 11 *in situ *component of DCIS-IDC samples, and 10 IDC samples; these served as initial sample sets. An additional 11 *in situ *component of DCIS-IDC samples were evaluated as an independent sample set. The non-neoplastic samples were obtained from perilesional mammary specimens from patients obtained during resection of benign lesions. A pathologist subjected all slides representative of pure DCIS to a careful histopathological analysis in order to ensure the absence of any previously undetected microinvasions. At least 5 years of follow-up data were available for all patients.

The patients had a mean age of 49 years, and none of them had received preoperative systemic treatment. The patients' clinical characteristics are summarized in Table 1. The expression patterns of estrogen receptor, progesterone receptor, and human epidermal growth factor receptor (HER)2/neu were positive in the majority of the samples. The research was approved by the Ethics Committee of the Medical and Research Center – Hospital A C Camargo, under protocol number 587/04. All participants gave written, informed consent.

**Table 1:** Patient and tumor characteristics

Specimen description	Stage	Age (years)	TNM	Nuclear grade	ER	PR	p53 status	HER2/neu
Initial sample								
43 DCIS (pure)	0	37	T3N0M0	ND	-	-	+	ND
44 DCIS (pure)	0	44	ToN0M0	2 and 3	+	+	-	+
46 DCIS (pure)	0	43	T2N0M0	3	+	+	-	+ (3+)
48 DCIS (pure)	0	52	T2N0M0	3	+	+	-	+ (3+)
49 DCIS (pure)	0	58	TicN0M0	3 (High grade)	-	-	-	+ (3+)
2 DCIS	IIa	48	T2N0M0	3	+	+	+	+
13 DCIS	IIa	75	T2N0M0	2	+	+	+	+ (2+)
25 DCIS	IIa	34	T2N0M0	3	+	+	+	+ (3+)
33 DCIS	I	38	T1cN0M0	3	+	-	-	-
45 DCIS	I	55	T1N0M0	3	-	-	+	+ (3+)
66 DCIS	IIIb	44	T4bN1M0	3	+	+	+	- (1+)
69 DCIS	IIb/IIIa	57	T2N1M0/pT2N2M0	2	+	+	ND	+ (2+)
75 DCIS	IIb	43	pT2N0M0	IDC 2/DCIS 3	-	-	+	+ (3+)
85 DCIS	I	73	T1cN0M0 pT3N0M0	2	+	+	-	+ (2+)
86 DCIS	ND	46	T2N1M0	3	+	+	+	+ (2+)
87 DCIS	IIa	48	T2N1M0	3	-	-	-	+ (2+)
1 IDC	IIa	45	T2N0M0	2	-	-	-	+ (3+)
3 IDC	IIa	43	T1cN0M0	3	+	-	-	+ (3+)
24 IDC	IIa	54	T2N0M0	3	+	+	+	+ (3+)
50 IDC	III b	71	T4N2M0	3	+	+	+	+ (2+)
51 IDC	II a	43	initial T2N0M0	3	-	ND	ND	+ (3+)
53 IDC	III a	43	T2N2M0	3	+	+	ND	+ (2+)
56 IDC	I	ND	ND	ND	ND	ND	ND	ND
80 IDC	IIa	44	pT1cpN1M0	3	+	+	-	-
81 IDC	IIb	31	T2N1M0	3	-	-	+	+ (3+)
83 IDC	IIa	48	T2N0M0	2	+	+	-	+ (2+)

Independent group of DCIS/IDC samples								
1 DCIS	ND	58	T1N0M0	3	+	+	+	+ (2+)
2 DCIS	ND	40	T1N1M0	3	+	+	-	+ (2+)
3 DCIS	ND	27	T1N0M0	1 (Low grade)	ND	ND	ND	ND
4 DCIS	ND	53	ND	3 (High grade)	+	+	+	+ (2+)
5 DCIS	ND	51	T1N0M0	2	+	+	ND	+ (2+)
6 DCIS	III a	54	T3N1M0	3 (High grade)	-	-	ND	ND
8 DCIS	ND	52	T2N1M0	3	-	-	+	+ (3+)
9 DCIS	ND	38	T1N1M0	3 (High grade)	-	-	+	- (1+)
3 DCIS	ND	39	T2N0M0	ND	+	+	+	-
8 DCIS	ND	49	T2N0M0	ND	+	+	ND	-
15 DCIS	II b	69	T2N1M0	ND	+	+	ND	+ (2+)

Unless otherwise stated, 'DCIS' means *in situ *component of DCIS-IDC. DCIS, ductal carcinoma *in situ*; DCIS-IDC, ductal carcinoma *in situ *with co-existing invasive ductal carcinoma; ER, estrogen receptor; IDC, invasive ductal carcinoma; ND, not determined; PR, progesterone receptor; TNM, tumor size, nodal status and metastasis.

### Laser capture microdissection

Cells were laser captured using the PixCell II LCM system (Arcturus Engineering, Mountain View, CA, USA). About 4,000 cells were captured from 4 to 7 μm frozen sections, mounted onto glass slides, and stained with 100 μl of nuclear fast red (C.I.60760; Certistain^®^; Merck, Darmstadt, Germany) for microscopy. Only one type of cells was isolated from each sample group. Non-neoplastic breast epithelium samples were epithelial cells without contamination with stromal cells; pure DCIS samples were tumor cells captured from ducts of pure DCIS lesion; *in situ *component of DCIS-IDC samples were tumor cells captured from *in situ *component of the DCIS-IDC lesion; and IDC samples were infiltrative tumor cells captured from the invasive lesion.

**Figure 1:**
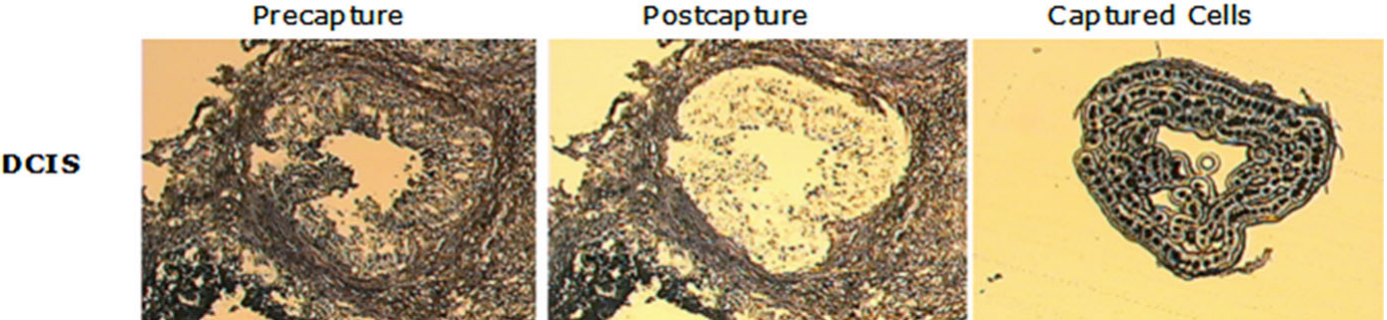
Breast epithelium captured from DCIS by LCM. Images of **(a) **pre-capture and **(b) **postcapture tissues, and **(c) **captured epithelial tumor cells. DCIS, ductal carcinoma *in situ*; LCM, laser capture microdissection.

A representative sample of cells from the pure DCIS depicting the different phases during the microdissection procedure is shown in Figure 1.

### RNA isolation and amplification

Laser capture microdissection (LCM) captured cells on CapSure™ HS LCM Caps (Arcturus Engineering) were resuspended in 10 μl of PicoPure RNA extraction buffer (Arcturus Engineering). Total RNA was extracted by using the PicoPure™ RNA Isolation kit (Arcturus Engineering #KT0204) and DNase treated using the RNase-Free DNase Set (Qiagen #79254; Qiagen-Germantown MD USA), in accordance with the manufacturer's instructions. A two-round linear amplification procedure, based on T7-driven amplification, was performed following a previously described protocol [23] with some modifications described below. The total RNA was first denatured at 70°C for 10 minutes in presence of 200 ng oligo (dT) [24]-T7 primer (5'-AAA CGA CGG CCA GTG AAT TGT AAT ACG ACT CAC TAT AGG CGC T (24)-3'; 57 base pairs) and snap cooled on ice.

Reverse transcription was performed by adding 1× first strand buffer and 0.01 mol/l dithiothrectol (Invitrogen Life Technology, Carlsbad, CA, USA), 2 μl diethylpyrocarbonate (DEPC; Sigma, St Louis, MO, USA) treated water, 40 U rRNasin (Promega, Madison, WI, USA), 1 mmol/l dNTP (Amersham Biosciences, Piscataway, NJ, USA), and 400 U SuperScript™ II Reverse Transcriptase (Invitrogen Life Technology) to a final volume of 20 μl. The reaction was incubated for 120 minutes at 42°C. Second-strand synthesis was performed by adding 53 μl of DEPC-treated water, 20 μl of 5× second strand buffer (Invitrogen Life Technology), 1 mmol/l dNTP, 1 U RNase H (Invitrogen Life Technology), 10 U *Escherichia coli *DNA ligase, and 40 U *E. coli *DNA polymerase I (Invitrogen Life Technology) to a final volume of 100 μl. The reaction was incubated for 2 hours at 16°C. Ten units of T4 DNA polymerase I (Invitrogen Life Technology) were added and incubated again at 16°C for 5 minutes.

The double strand cDNA (dscDNA) was stopped by adding 0.05 mol/l EDTA. UltraPure™ Phenol (Invitrogen, Carlsbad, CA, USA):chloroform:isoamyl alcohol (Merck), at a ratio of 25:24:1 and a pH of 8.0, was used for cDNA purification. The dscDNA was precipitated with absolute ETOH (Merck) and resuspended in 10 μl DEPC-treated water. The dscDNAs were subjected to *in vitro *transcription using reagents from Ribomax™ Large scale RNA production system T7 kit (Promega), in accordance with the manufacturer's recommendation. The amplified RNA (aRNA) was reverse transcribed into cDNA using 9 μg random hexamer (dN6; Amersham Biosciences, Little Chalfont, UK). cDNA synthesis was continued with the same conditions used in the first strand of the first round. The second strand was synthesized using Advantage^® ^cDNA Polymerase (Clontech, Mountain View, CA, USA), and purification was performed in accordance with the methodology cited above.

The aRNA quality, in terms of purity and integrity, was assessed by absorbance at 260/280 nm using a GeneQuant *pro *spectrophotometer (Amersham Pharmacia Biotech, Little Chalfont, UK) and by electrophoresis in 1% UltraPure™ Agarose (Invitrogen Life Technology) gel with ethidium bromide staining (Sigma, St Louis, MO, USA), respectively. Only aRNA samples yielding a minimum of 15 μg and presenting a smear concentration between 300 and 700 base pairs (which guarantees high quality hybridization) were further processed. Total RNA from HB4a normal luminal epithelial mammary cells [24] was extracted and amplified following the same protocol and used as a reference for microarray hybridizations.

### cDNA microarrays and probes

We used a customized cDNA platform (4.8K002 platform) comprising 4,608 cDNAs that represent human genes [25]. The labeled cDNA was generated in a reverse transcriptase reaction in the presence of 4 μg aRNA, 9 μg random hexamer primer (Invitrogen Life Technology), Cy3-labeled or Cy5-labeled dCTP (Amersham Biosciences, Little Chalfont, UK), and 400 U SuperScript II (Invitrogen Life Technology). The residual dye was removed using illustra AutoSeq™ G-50 (GE Healthcare, Little Chalfont, UK). Equal amounts of test and reference cDNA reverse colored Cy-labeled product were competitively hybridized against the cDNA probes in microarray slides. Dye swap was performed for each sample analyzed and used as replicate samples. Pre-hybridization was carried out in a humidified chamber at 42°C for 16 to 20 hours, and hybridizations were performed on GeneTac Hybridization Station (Genome Solutions, Ann Arbor, MI, USA) at 42°C.

### Intensity signal capture and analysis

After hybridization, slides were washed as follows: 2× Saline-Sodium Citrate (SSC) for 10 minutes, 0.1 × SSC/0.1% SDS for 10 minutes (two times), and 0.1 × SSC for 10 minutes (two times) at 37°C. All solutions were pre-heated to 42°C. Hybridized arrays were scanned on the ScanArray™ Express (Packard BioScience Biochip Technologies, Billerica, MA, USA), and Cy5/Cy3 signals were quantified using the histogram method with ScanArray Express software (Perkin-Elmer Life Sciences, Boston, MA, USA). Fluorescent intensities of Cy5 and Cy3 channels on each slide were subjected to spot filtering and normalization. We first eliminated all saturated points (≥ 63,000; approximately 16 bits) and performed a local background subtraction, considering for analysis only those spots with positives values. Normalization was performed using locally weighted linear regression within and across arrays for inter-slide normalization. Local normalization has the advantage that it can help to correct for systematic spatial variations in the array [26]. After normalization, data for each gene were reported as the logarithm of the expression ratio used to represent the relative gene expression levels in the experimental samples. The raw data from hybridizations and experimental conditions can be obtained at the Gene Expression Omnibus [27] under accession number GSE11042. A detailed description of the platform array is available in accession number GPL1930.

### Statistical analysis

For applying the concept for molecular divergence and to identify the most distinct group of samples, the general expression patterns were compared between the sample groups (non-neoplastic, pure DCIS, *in situ *component of DCIS-IDC, and IDC) using the analysis of variance (ANOVA) statistical test (positive false discovery rate – pFDR < 0.01) [28] followed by Tukey's test [29]. To select the putative genes involved in DCIS progression, two-by-two comparisons among non-neoplastic, pure DCIS, and *in situ *component of DCIS-IDC sample groups were performed; differentially expressed genes, whose expression levels exhibited at least twofold change, were selected. Downregulated and upregulated genes were analyzed separately. A Venn diagram was constructed to select the genes of interest.

To determine decay rate statistics for collections of genes belonging to the different gene sets, automated analysis of gene function was required. The functional assignments of the genes were obtained using the χ^2 ^distribution of Onto-Tools (developed by Intelligent Systems and Bioinformatics Laboratory from Department of Computational Sciences of Wayne State University) [30]. Functional processes were considered as significant if the *P *value was under 0.01. Functional assessments, which were represented by only one gene in the platform, were not taken into consideration in order to avoid artifactual results.

For clustering samples based on gene expression profiles, we applied a nonsupervised hierarchical clustering based on Euclidean distance and average linkage. The reliability of the clustering was assessed by the Bootstrap technique using MEV (MultiExperiment Viewer – Boston, MA, USA) [31].

### Quantitative RT-PCR

Quantitative RT-PCR reactions were performed using the ABI Prism™ 7700 Sequence Detection System (Applied Biosystems, Foster City, CA, USA). Aliquots of cDNA from aRNA were used as templates. RT-PCR reactions were carried out using SYBR^® ^Green PCR MasterMix (Applied Biosystems) in a total volume of 20 μl using the following program: 2 minutes at 50°C and 10 minutes at 95°C for the initial denaturing, followed by 40 cycles at 95°C for 15 seconds and 60°C for 1 minute. The list of oligonucleotide sequences is shown in Table 2. Finally, dissociation curves were generated at 95°C for 15 seconds, 60°C for 30 seconds, and 95°C for 15 seconds. To evaluate the amplification of nonspecific products and primer-dimer formation, dissociation curves were analyzed and aliquots of each reaction were subjected to silver staining acrylamide gel electrophoresis. The efficiency of each pair of primers was calculated using standard curve dilutions (as described in the Applied Biosystems protocols). The analysis was conducted using the cDNA converted from the aRNA used in the microarray study. The reactions were performed in duplicate. Three internal control genes, namely *HPRT1 *[GenBank:NM_000194] [32], *GAPDH *[GenBank:AJ005371], and *BCR *[GenBank:NM_004327], were considered in gene expression normalization. Relative gene expression between sample groups was calculated using the Pfaffl model [33], employing the efficiency-corrected equation.

**Table 2:** List of the oligonucleotides sequences used for quantitative real-time PCR

	Gene symbol	Annotation	Primer sequences
Target genes	*CGI-41*	CGI-41 protein	Forward: CCAGGCGTGCAGGGTATC Reverse: GCCCCCGCTGCACAT
	
	*C16orf5*	Chromosome 16 open reading frame 5	Forward: CAGCCAGAGCAGTTAGCCAGTTA Reverse: CTGACTCCAGACAACTTACCCATTC
	
	*GOSR2*	Golgi SNAP receptor complex member 2	Forward:GCAGGAGAGACAGCGAGAAGA Reverse:TGCAGTGATTCGTCCATTGG
	
	*MARK3*	MAP/microtubule affinity-regulating kinase 3	Forward: AGACACTCAGTGATTCAGAATGGC Reverse: GAAGCAACTGGAGTTCTCTGATCA
	
	*LOX*	Lysyl oxidase	Forward: CAGGACATCATGCGTATGCC Reverse: CCAGGCACTGATTTATCCATTG
	
	*STK25*	Serine/threonine kinase 25 (STE20 homolog, yeast)	Forward: ACCTGGTGGAGCGAGTGC Reverse: TTCAGCGGGTGGATGTCAG
	
	*SULF-1*	Sulfatase 1	Forward:GGCATTTTGAATCAGCTACACGTA Reverse:TCCCATCCATCCCATAACTGTC
	
	*TXNL2*	Thioredoxin-like 2	Forward: GACCACAGGCGTGCACC Reverse: GATACCTTTCCTCATCCATCACAAG

Endogenous genes	*HPRT*	hypoxanthine phosphoribosyltransferase 1	Forward: GAACGTCTTGCTCGAGATGTGA Reverse: TCCAGCAGGTCAGCAAAGAAT
	
	*GAPDH*	Glyceraldehyde 3-phosphate dehydrogenase	Forward: ACCCACTCCTCCACCTTTGA Reverse: CTGTTGCTGTAGCCAAATTCGT
	
	*BCR*	Breakpoint cluster region	Forward: CCTTCGACGTCAATAACAAGGAT Reverse: CCTGCGATGGCGTTCAC

## Results

### Laser microdissection, RNA extraction, and microarray experiments

To generate a precise correlation between specific epithelial cells and their gene expression patterns, we integrated the use of LCM and T7-based RNA amplification with cDNA microarrays. This procedure permitted gene expression profile analysis with a cell based, rather than tissue based, resolution. To characterize the molecular alterations of cells from the *in situ *component of the two breast cancer lesions, namely DCIS and DCIS-IDC, we analyzed the gene expression profiles of both. We also used non-neoplastic epithelial cells and cells from IDC lesions as examples of zero and complete progression, respectively.

The Pearson correlation average between all hybridization replicates used in this study was 0.91. All replicates clustered together in dendrograms reporting suitable correction of the individual dye incorporation efficiency by normalization procedure and high experimental reproducibility.

### Molecular divergence concepts based on general cell-based gene expression profile

To classify the groups of samples according to their molecular divergence, we used the number of differentially expressed genes as our distance measure; the larger the number of differentially expressed genes between one sample type and all the others, the more distant the sample was allocated and consequently more molecularly divergent. To accomplish this, an ANOVA test corrected by pFDR (<0.01) was performed on the four sample groups, identifying 764 differentially expressed genes (see Additional file 1). Next, Tukey's test was performed through two by two comparisons between the distinct groups. As expected, the non-neoplastic epithelial breast cells exhibited the most divergent expression profile (29%). A total of 221 genes out of 764 were only differentially expressed between non-neoplastic and the three tumor cell groups. In contrast, for cells from pure DCIS, the *in situ *component of DCIS-IDC and IDC, when compared with all of the other three, there were 12 (1.6%), 37 (4.8%) and 6 (0.3%) differentially expressed genes out of 764, respectively.

**Figure 2:**
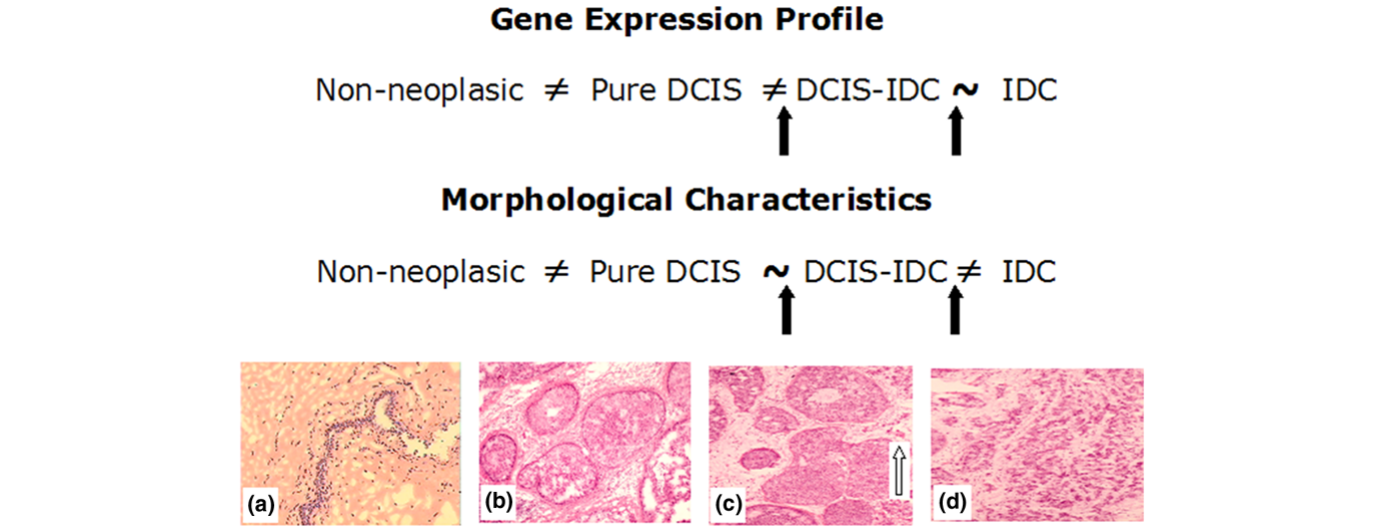
Comparison among the gene expression profile and morphological characteristics of progression of breast ductal carcinoma. **(a) **Non-neoplastic tissue (20×). **(b) **Pure DCIS (10×). **(c) **DCIS-IDC (arrow; hand lens). **(d) **IDC (hand lens). DCIS, ductal carcinoma *in situ*; DCIS-IDC, ductal carcinoma *in situ *with co-existing invasive ductal carcinoma; IDC, invasive ductal carcinoma.

Using the same metrics, a new round of analysis was performed excluding the non-neoplastic sample group in order to identify which tumor cell population exhibits the highest molecular divergence level among the three analyzed groups. The ANOVA corrected by pFDR (<0.01) identified 90 variable genes among the three groups of neoplastic cells (see Additional file 2). Tukey's test revealed that cells from pure DCIS exhibited the most distinct gene expression profile, with 75 genes (83%) out of 90 being differentially expressed in comparison with the other two groups of tumor cells. For cells from the *in situ *component of DCIS-IDC and cells from IDC lesions, 16 genes (18%) and 6 genes (7%) out of 90 were found to be differentially expressed, respectively (Figure 2). A parallel analysis between molecular and morphological aspects yielded contradictory results, because – in terms of morphological features – IDC had the most distinct features and pure DCIS and the *in situ *component of DCIS-IDC lesions exhibited identical patterns. On the other hand, in terms of molecular features, cells from pure DCIS exhibited the most distinct molecular expression profile, and consequently cells from *in situ *component of DCIS-IDC retains high similarity to cells from IDC, which suggests alterations in gene expression programs of cells from *in situ *component of DCIS-IDC before their progression to IDC.

### Selection of putative genes involved in ductal carcinoma progression

DCIS progresses to malignant disease when some of the tumor cells acquire invasive capacity. Our previous data strongly suggested that the *in situ *component of DCIS-IDC already harbors molecular alterations that signal establishment of the invasive process. Based on these findings, we reasoned that the genes potentially involved in the earliest molecular step in acquiring the capacity to invade the surrounding tissue might be among the genes that are differentially expressed between cells from *in situ *component of DCIS-IDC and both pure DCIS and non-neoplastic groups.

**Figure 3:**
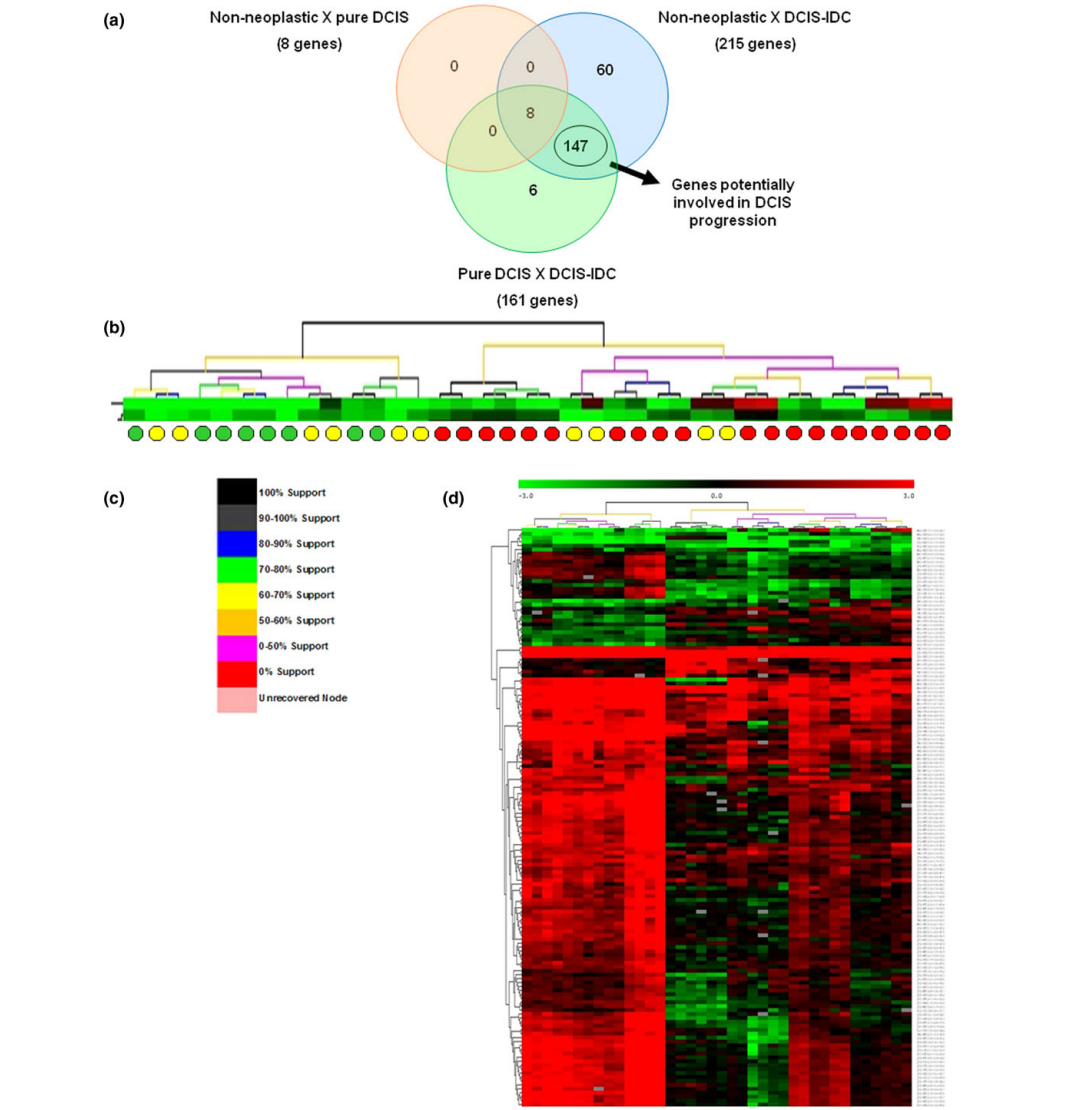
Putative genes involved in ductal carcinoma progression. **(a) **Venn diagram depicting the common and distinct genes in each comparison (downregulated and upregulated genes were analyzed separately). **(b) **Dendogram based on the expression profile of the 147 gene set. Green circles indicate non-neoplastic samples; yellow indicates pure DCIS, and red indicates *in situ *component of DCIS-IDC samples. **(c) **Legend of cluster support. **(d) **Scaled down representation of the entire cluster shown in panel b. Each row represents a single gene and each column a sample. Red indicates upregulation, green indicates downregulation, and black indicates no change in expression level compared with the reference sample. Gray indicates that no intensity was detected. DCIS, ductal carcinoma *in situ*; DCIS-IDC, ductal carcinoma *in situ *with co-existing invasive ductal carcinoma.

To identify these genes we conducted an ANOVA test corrected by pFDR (<0.01) among non-neoplastic, pure DCIS, and the *in situ *component of DCIS-IDC, identifying 785 altered genes (see Additional file 3). The two-by-two comparisons between samples from the three groups revealed 8, 215, and 161 genes between non-neoplastic versus pure DCIS, non-neoplastic versus *in situ *component of DCIS-IDC, and pure DCIS versus the *in situ *component of DCIS-IDC, respectively (Figure 3a; see Additional file 4). To eliminate genes involved in tumor formation from those potentially involved in tumor progression, we subtracted eight genes that were differentially expressed between cells from non-neoplastic and pure DCIS cells (Figure 3a). The common differentially expressed genes between *in situ *component of DCIS-IDC versus non-neoplastic or pure DCIS were selected in order to choose the more robust genes, yielding 147 genes classified as potentially implicated in DCIS progression. From those, 126 were upregulated and 21 were downregulated in pure DCIS (see Additional file 5), suggesting that the malignant process of tumor cells from pure DCIS to *in situ *component of DCIS/IDC occurs rather by downregulation than by upregulation of gene expression.

Functional annotation of this gene set, according to the Onto-Tools database, revealed a statistically significant enrichment of genes involved in cell adhesion (represented by *C20orf42 *[GenBank:AL118505], *LPXN *[GenBank:NM_004811], *PCLKC *[GenBank:NM_017675], *DGCR2 *[GenBank:NM_005137], *AZGP1 *[GenBank:NM_001185], *CHST10 *[GenBank:NM_004854], *ITGB2 *[GenBank:NM_000211], *PLEKHC1 *[GenBank:AK291738], *PCDH10 *[GenBank:NM_032961], and *NEDD9 *[GenBank:NM_006403]) and cellular defense (represented by *CXCL9 *[GenBank:NM_002416], *MAPRE2 *[GenBank:NM_014268], and *C3AR1 *[GenBank:NM_004054]). *LPXN *[GenBank:NM_004811] and *NEDD9 *[GenBank:NM_006403] have been reported as being involved in cancer progression. Over-expression of *LPXN *[GenBank:NM_004811] and *NEDD9 *[GenBank:NM_006403] resulted in increased migration in the bone-derived metastatic prostate cancer cell line [34] and in promotion of metastatic melanoma [35], respectively. These reports support our data, which reveal high expression of these genes in the *in situ *component of DCIS-IDC compared with pure DCIS cells. The 147 genes were organized into seven different biological classes in a hierarchical manner (Table 3).

**Table 3:** Genes potentially implicated in DCIS progression functionally classified within the biological process category

Functional process	Downregulated genes in pure DCIS	Upregulated genes in pure DCIS
Cell adhesion and migration	-	*AZGP1*, *C20orf23*, *C20orf42*, *CHST10*, *COL17A1*, *DGCR2*, *GPR98*, *ITGB2*, *KIF1A*, *LPXN*, *NEDD9*, *PCDH10*, *PCLKC*, *PLEKHC1*, *RGMB*

Signal transduction	*CORO1C*, *CXCL9*, *IGSF6*, *LOX*, *NCOA4*, *NMU*, *SKIL*	*ARHGAP19*, *ARHGAP9*, *C16orf5*, *C3AR1*, *CHRNB1*, *EPOR*, *FCGR2B*, *FCN1*, *FGFBP1*, *GIPC1*, *GPR77*, *KDR*, *MAPRE2*, *PIAS2*, *RHOU*, *STK25*

Cell proliferation and apoptosis	*NOX4*, *SULF1*	*ANAPC13*, *CDC45L*, *ERC1*, *IFT57*, *RARRES3*, *REC8L1*, *SHC1*, *UTP20*

Transcriptional regulation	*MED10*, *PHTF1*	*AOF2*, *ATF2*, *ETNK2*, *IRF8, MBD3*, *MGC21874*, *SMARCA3*, *SOX13*, *TARDBP*, *ZBTB5*

Metabolism	*P4HA1*	*B4GALT5*, *BCHE*, *CA3*, *CPNE3*, *CPT1A*, *DHRS12*, *FN3K*, *GBGT1*, *OSBPL7*, *PEPD*, *PITPNM2*, *UFD1L*, *ZFP36L1*

Miscellaneous	*CCT5*, *MARCH8*, *PTBP2*, *RAD51AP1*	*ALMS1*, *ARFIP1*, *BOP1*, *CAMP*, *CAV1*, *CIRBP*, *CLINT1*, *CLTCL1*, *CTSZ*, *DHX35*, *EYA2*, *FCGR3A*, *GOSR2*, *IMMT*, *INOC1*, *KBTBD10*, *KCTD15*, *KIAA0664*, *KPNA6*, *LSM4*, *MARK3*, *MRPS17*, *NGDN*, *NUP50*, *P4HB*, *PMPCA*, *POLD3*, *POMGNT1*, *PPP2R3A*, *RABEPK*, *RPL3*, *RPL41*, *RSL1D1*, *SAMD4A*, *SLC6A20*, *SLC9A5*, *SPOCK2*, *STX11*, *SV2B*, *SYN1*, *TBC1D9B*, *TRAP1*, *TXNDC11*, *TXNL2*, *UBXD1*, *VPS54*

Unknown	*C13orf23*, *C7orf24*, *HN1*, *KIAA1211*, *RUNDC1*	*ADFP*, *ANKRD6*, *BIN2*, *C10orf26*, *C13orf24*, *C1orf66*, *CTTNBP2NL*, *DENND3*, *FAM40B*, *ITPKC*, *KIAA0748*, *LETMD1*, *LRCH2*, *LTBP3*, *NCDN*, *PPM1H*, *PPTC7*, *RNF43*

Genes belonged to more than one biological process were assigned in a hierarchical manner in the following order: cell adhesion and migration; signal transduction; cell proliferation and apoptosis; transcriptional regulation; and metabolism. Those with no classification in the five categories were classified as miscellaneous. DCIS, ductal carcinoma *in situ*.

The hierarchical clustering based on the expression pattern of this gene set resulted in two main branches with high level of reliability (Figure 3b,c). Non-neoplastic samples and 60% of the pure DCIS samples were discriminated from 100% of the *in situ *component of DCIS-IDC samples (Figure 3b). The ability of this gene set to generate an expression pattern (Figure 3d) that segregated the two groups of cells (pure DICS and *in situ *component of DCIS-IDC) relatively well, grouping together the samples representing cells from non-neoplastic and cells from pure DCIS lesion, strengthened the hypothesis that those genes might be involved in the early molecular alterations that are necessary for launching of the invasive process.

### Confirmation of differential expression between pure DCIS and DCIS-IDC by quantitative RT-PCR

In order to evaluate the robustness of our microarray findings and to avoid choosing false differentially expressed genes because of technical limitations, we randomly selected eight genes from the 147 gene set (Table 4) and performed quantitative RT-PCR in duplicate experiments. The average of the internal control genes (*HPRT1 *[GenBank:NM_000194], *GAPDH *[GenBank: AJ005371] and *BCR *[GenBank:NM_004327]) was used in a normalization procedure.

**Table 4:** Genes selected for quantitative RT-PCR experiments

	Gene symbol	Microarray (fold change)	Quantitative RT-PCR (fold change)
Target genes	*CGI-41*	5.3	1.9
	
	*C16orf5*	5.5	2.6^a^
	
	*GOSR2*	4.0	2.9^a^
	
	*MARK3*	4.9	-1.4
	
	*LOX*	-2.0	-11.6^a^
	
	*STK25*	2.0	-1.5
	
	*SULF-1*	-4.0	-11.9^a^
	
	*TXNL2*	4.2	3.0^a^

Positive and negative numbers indicate upregulated and downregulated genes in pure DCIS, respectively. ^a^Genes confirmed using both methodologies. DCIS, ductal carcinoma *in situ*.

Using the initial sample sets and the criteria of ≥ 2-fold change, five out of eight genes (62.5%) exhibited agreement in both methodologies. The genes *C16orf5 *[GenBank:NM_013339], *GOSR2 *[GenBank:NM_004287], and *TXNL*2 [GenBank:AL138831] were upregulated, and *LOX *[GenBank:NM_002317] and *SULF-1 *[GenBank:NM_001128206] were downregulated in pure DCIS in comparison with *in situ *component of DCIS-IDC. These five genes were also evaluated in an independent group of 11 *in situ *component of DCIS-IDC samples (Table 5). Three genes (60%; *GOSR2 *[GenBank:NM_004287], *LOX *[GenBank:NM_002317], and *SULF-1 *[GenBank:NM_001128206]) exhibited agreement with the previous data, strengthening the hypothesis that these genes are involved in the malignant process of DCIS.

**Table 5:** Genes selected for quantitative RT-PCR experiments using an independent sample set of *in situ *component of DCIS/IDC (11 samples)

	Gene symbol	Microarray (fold change)	Quantitative RT-PCR (fold change)
Target genes	*C16orf5*	5.5	1.4
	
	*GOSR2*	4.0	6.4^a^
	
	*LOX*	-2.0	-3.6^a^
	
	*SULF-1*	-4.0	-37^a^
	
	*TXNL2*	4.2	-13.4

Positive and negative numbers indicate upregulated and downregulated genes in pure DCIS, respectively. ^a^Genes confirmed using both methodologies. DCIS, ductal carcinoma *in situ*; IDC, invasive ductal carcinoma.

**Figure 4:**
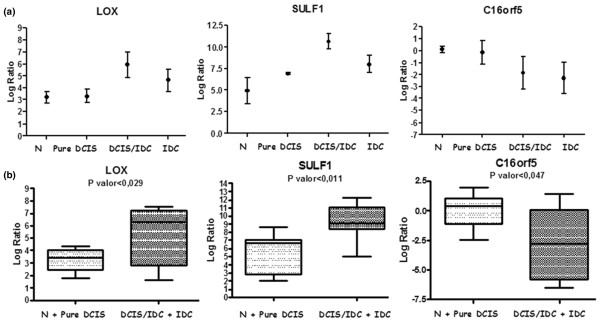
Gene expression behavior among samples that mimic breast cancer progression. **(a) **Gene expression difference between identical morphologic samples (pure DCIS and *in situ *component of DCIS-IDC) by analysis of variance. **(b) **Comparison between two groups (non-neoplastic [N] + pure DCIS and *in situ *component of DCIS-IDC + IDC] by quantitative RT-PCR. DCIS, ductal carcinoma *in situ*; DCIS-IDC, ductal carcinoma *in situ *with co-existing invasive ductal carcinoma.

Because we were interested in identifying genes that are involved with the acquisition of invasive capacity in DCIS, we reasoned that these genes should not exhibit differential expression between cells from non-neoplastic cells and pure DCIS cells, as neither of these cell types has established invasion capacity. Likewise, we would expect there to be no difference in expression level between cells from the *in situ *component of DCIS-IDC and IDC lesions, in which – despite of the morphological differences in the tissue – the capacity and program of invasion are already established. Therefore, the five genes confirmed in the initial sample set were evaluated in non-neoplastic and IDC samples. Based on our selection criteria, three genes (*C16orf5 *[GenBank:NM_013339], *LOX *[GenBank:NM_002317], and *SULF-1 *[GenBank:NM_001128206]) showed the expected gene expression behavior in the four sample groups, exhibiting slight or no difference between the group with no invasive program (non-neoplastic and pure DCIS) and that in which the invasive program is already established (DCIS-IDC and IDC; Figure 4a). The expression levels of these three genes exhibited statistically significant differences between the cells lacking the invasive program (non-neoplastic and pure DCIS) and cells possessing the invasive program (*in situ *component of DCIS-IDC and IDC; Figure 4b). This strongly indicates that *LOX *[GenBank:NM_002317] and *SULF1 *[GenBank:NM_001128206], confirmed in the initial and independent group of *in situ *component of DCIS-IDC samples, are potentially involved in the acquisition of invasive capacity in DCIS.

## Discussion

Characterization of the molecular events that are associated with DCIS progression has been among the major aims of the scientific community. Even though great efforts to decipher the molecular basis of DCIS have been made [2,9,13,14,16,36-38], these molecular events remain poorly understood. Studies have been limited by the low availability of pure DCIS frozen samples. In this study, we combined the use of LCM, RNA amplification, and microarray technology to characterize the gene expression pattern of cells captured from pure DCIS and *in situ *component of DCIS-IDC, which retain identical morphological characteristics in the tissue. Non-neoplastic epithelial cells and IDC lesion cells were also analyzed.

The gene expression profile analysis of cells from three types of breast cancer lesions (pure DCIS, *in situ *component of DCIS-IDC, and IDC) yielded surprising results. We found that, rather than cells from IDC, cells from pure DCIS had the most divergent molecular aspects, which is in contrast to the morphological features. This finding is directly and indirectly supported by recent reports. Recent studies relating the expression of Her-2/neu, steroid receptors (estrogen receptor and progesterone receptor), Ki67, p53, and epidermal growth factor receptor in pure DCIS to *in situ *component of DCIS-IDC have suggested that both components have distinct molecular characteristics [39-42]. Gene expression analyses of the two distinct morphological components, the *in situ *component of DCIS-IDC and IDC, using matched and non-matched samples have identified very similar molecular profiles [2,9,16,38]. Based on these findings, we speculate that either the acquisition of invasive capacity of the DCIS cells is driven by a very small number of genes that play a key role in the invasion process, or the molecular alteration occurs before the morphological modification of the lesion. The findings presented here support the latter hypothesis, strongly suggesting that the molecular alteration of cells from *in situ *component of DCIS-IDC is already established before the lesion exhibits morphological changes.

In practical terms, one of the major contributions of our study lies in using the molecular divergences between *in situ *components of the two types of lesions, which have identical morphological characteristics but distinct malignant potential, to identify gene markers that may predict the risk for progression from pure DCIS to invasive disease.

We validated five genes by quantitative RT-PCR in the initial sample sets, which showed 62.5% of agreement in both methodologies and ensured that our microarray data were robust. The *GOSR2 *[GenBank:NM_004287], *C16orf5 *[GenBank:NM_013339], and *TXNL2 *[GenBank:AL138831] genes were over-expressed in pure DCIS when compared with the *in situ *component of DCIS-IDC. The *GOSR2 *[GenBank:NM_004287] finding was also confirmed in an independent group of 11 *in situ *component DCIS-IDC samples. The *GOSR2 *[GenBank:NM_004287] gene encodes a membrane trafficking protein, which transports proteins among the medial- and trans-Golgi compartments and is involved in signal transduction and transporter activity. *C16orf5 *[GenBank:NM_013339] was confirmed in the initial set of samples, and in an independent set it exhibited the same tendency in relative expression level (1.4-fold change) but was eliminated by the cut-off adoption criterion (fold change > 2). *C16orf5 *[GenBank:NM_013339] has an uncommonly high content of proline residues (40% over 104 residues) at the amino-terminus of the protein and is highly expressed in brain [43]. This gene is also known as *CDIP *(cell death-inducing protein), a potential apoptosis inducer that is associated with caspase-8 cleavage, implicating the extrinsic cell death pathway in apoptosis mediated by *CDIP *[44]. The over-expression of *TXNL2 *[GenBank: AL138831] in pure DCIS appears to be dependent on the initial set of samples, because in the independent group of samples we found the change in its expression to be in the opposite direction (under-expression).

*LOX *[GenBank:NM_002317] and *SULF-1 *[GenBank:NM_001128206] were over-expressed in the *in situ *component of DCIS-IDC when compared with pure DCIS in both initial and independent sample sets. The *LOX *[GenBank:NM_002317] gene mediates metastasis of human breast cancer cells in a mouse model [45] and regulates *in vitro *breast cancer cell migration and cell-matrix adhesion through the regulation of Scr kinases and FAK [46], making it a candidate for predicting progression of DCIS. Other recent studies showed that *LOX *[GenBank:NM_002317] expression correlates positively with tumor progression and co-localization with hypoxic regions (defined by hypoxia inducible factor-1α expression) in DCIS and IDC primary tumors [47]. The gene *SULF-1 *[GenBank:NM_001128206] modulates heparin-binding growth factor signaling, and diminishes proliferation and mitogenecity *in vitro *in head and neck squamous carcinoma [48]. Evaluation of *SULF-1 *[GenBank:NM_001128206] expression levels in primary invasive breast tumors by RNA *in situ *hybridization indicated that this gene is down-regulated in the majority (60%) of samples, with a predominant association with lobular histology [49], which is a disagreement with our data. However, in human pancreatic adenocarcinoma tumors this gene is upregulated, and it is widely expressed in the human pancreatic adenocarcinoma cell line [50]. Despite incomplete concordance among our data and data from other groups for the *SULF*-*1 *[GenBank:NM_001128206] gene, over-expression in the *in situ *component of DCIS-IDC and IDC samples was unequivocal. Moreover, *SULF-1 *[GenBank:NM_001128206] and *LOX *[GenBank:NM_002317] exhibited statistically significant differences when the samples were grouped based on invasive behavior (non-neoplastic plus pure DCIS and *in situ *component of DCIS-IDC plus IDC), suggesting their putative involvement with the malignant transformation of DCIS.

The findings of the present study might have been influenced by the small number of pure DCIS, which retain very specific characteristic, such as high grade and HER2 positivity. Unfortunately, this sample group, because of difficulty in obtaining fresh tissue from this type of lesion, could not be evaluated as an independent group. Therefore, the actual role of these candidate genes in the malignant process of DCIS requires further investigation. Nevertheless, the evidence presented in this study identifies these genes as potential candidates for predicting risk for progression of pure ductal carcinoma.

## Conclusions

Our findings strongly suggest that the cells from DCIS with the potential to become invasive exhibit modifications in the general gene expression pattern before the morphological alteration of lesion becomes visible. The *SULF-1 *[GenBank:NM_001128206] and *LOX *[GenBank:NM_002317] genes are candidate molecular markers that may be used to predict the risk for DCIS progression.

## Abbreviations

ANOVA: analysis of variance; aRNA: amplified RNA; DCIS: ductal carcinoma *in situ*; DCIS-IDC: ductal carcinoma *in situ *with co-existing invasive ductal carcinoma; DEPC: diethylpyrocarbonate; dscDNA: double strand cDNA; HER: human epidermal growth factor receptor; IDC: invasive ductal carcinoma; LCM: laser capture microdissection; RT-PCR: reverse transcription polymerase chain reaction.

## Competing interests

The authors declare that they have no competing interests.

## Authors' contributions

NC performed all experiments and wrote the manuscript. CO reviewed the histological specimens and performed the LCM. CT carried out the bioinformatics analysis of the microarray data. EB participated in acquisition of microarray data. MM contributed with the breast cancer specimens and discussions. FS supervised the histological analysis and discussed the study design. HB supervised the bioinformatics analysis, helped in designing the study, and revised the manuscript. DC designed the study, interpreted the data, and wrote the manuscript. All authors read and approved the final manuscript.

## Supplementary Material

Additional file 1 Differentially expressed genes: non-neoplastic, pure DCIS, *in situ *component of DCIS-IDC, and IDC lesions. Presented is a table listing the differentially expressed genes among cells from non-neoplastic, pure DCIS, *in situ *component of DCIS-IDC, and IDC lesions. Click here for file

Additional file 2 Differentially expressed genes: pure DCIS, *in situ *component of DCIS-IDC, and IDC lesions. Presented is a table listing the differentially expressed genes among tumor cells from pure DCIS, *in situ *component of DCIS-IDC, and IDC lesions. Click here for file

Additional file 3 Differentially expressed genes: non-neoplastic, pure DCIS, and *in situ *component of DCIS-IDC lesions. Presented is a table listing the differentially expressed genes among cells from non-neoplastic, pure DCIS, and *in situ *component of DCIS-IDC lesions. Click here for file

Additional file 4 Differentially expressed genes resulting from of 2 × 2 comparisons. Presented is a table listing the differentially expressed genes resulting from of 2 × 2 comparisons: non-neoplastic × pure DICS; non-neoplastic × *in situ *component of DCIS-IDC; and pure DCIS × *in situ *component of DCIS-IDC. Click here for file

Additional file 5 147 Genes differentially expressed between pure DCIS and *in situ *component of DCIS-IDC. Presented a table listing the 147 genes differentially expressed between pure DCIS and *in situ *component of DCIS-IDC with the respective fold changes. Click here for file
