# Supplementary material for: Evidence that molecular changes in cells occur before morphological alterations during the progression of breast ductal carcinoma
Source: Breast Cancer Res. 2008 Oct 17;10(5):R87. doi: 10.1186/bcr2157 (PMC2614523; doi:10.1186/bcr2157)
Supplement: Additional file 1 — Differentially expressed genes: non-neoplastic, pure DCIS, in situ component of DCIS-IDC, and IDC lesions. Presented is a table listing the differentially expressed genes among cells from non-neoplastic, pure DCIS, in situ component of DCIS-IDC, and IDC lesions. [file bcr2157-S1.pdf]

Additional Data File 1 - 764 genes

Comparison among Non-neoplastic cells, pure DCIS, *in situ* component of DCIS-IDC

GeneID	Symbol	Description
51393	TRPV2	transient receptor potential cation channel, subfamily V, member 2
64284	RAB17	RAB17, member RAS oncogene family
58533	SNX6	sorting nexin 6
6590	SLPI	secretory leukocyte peptidase inhibitor
58472	SQRDL	sulfide quinone reductase-like (yeast)
23321	TRIM2	tripartite motif-containing 2
23203	PMPCA	peptidase (mitochondrial processing) alpha
8031	NCOA4	nuclear receptor coactivator 4
26000	TBC1D10B	TBC1 domain family, member 10B
29965	C16orf5	chromosome 16 open reading frame 5
23351	KIAA0323	KIAA0323
7738	ZNF184	zinc finger protein 184
4704	NDUFA9	NADH dehydrogenase (ubiquinone) 1 alpha subcomplex, 9, 39kDa
26509	FER1L3	fer-1-like 3, myoferlin (C. elegans)
56957	OTUD7B	OTU domain containing 7B
9079	LDB2	LIM domain binding 2
160760	PPTC7	PTC7 protein phosphatase homolog (S. cerevisiae)
3791	KDR	kinase insert domain receptor (a type III receptor tyrosine kinase)
23048	FNBP1	formin binding protein 1
23048	FNBP1	formin binding protein 1
51187	C15orf15	chromosome 15 open reading frame 15
7922	SLC39A7	solute carrier family 39 (zinc transporter), member 7
54482	CCDC76	coiled-coil domain containing 76
1852	DUSP9	dual specificity phosphatase 9
56474	CTPS2	CTP synthase II
55777	MBD5	methyl-CpG binding domain protein 5
23451	SF3B1	splicing factor 3b, subunit 1, 155kDa
51061	TXNDC11	thioredoxin domain containing 11
23225	NUP210	nucleoporin 210kDa
29997	GLTSCR2	glioma tumor suppressor candidate region gene 2
6397	SEC14L1	SEC14-like 1 (S. cerevisiae)
84928	FLJ14803	hypothetical protein FLJ14803
54894	RNF43	ring finger protein 43
1475	CSTA	cystatin A (stefin A)
9806	SPOCK2	sparc/osteonectin, cwcv and kazal-like domains proteoglycan (testican) 2
55577	NAGK	N-acetylglucosamine kinase
79157	ET	hypothetical protein ET
23586	DDX58	DEAD (Asp-Glu-Ala-Asp) box polypeptide 58

8666	EIF3S4	eukaryotic translation initiation factor 3, subunit 4 delta, 44kDa
114112	TXNRD3	thioredoxin reductase 3
4640	MYO1A	myosin IA
8880	FUBP1	far upstream element (FUSE) binding protein 1
6574	SLC20A1	solute carrier family 20 (phosphate transporter), member 1
1456	CSNK1G3	casein kinase 1, gamma 3
79053	ALG8	asparagine-linked glycosylation 8 homolog (S. cerevisiae, alpha-1,3-glucosyltransferase)
10483	SEC23B	Sec23 homolog B (S. cerevisiae)
4259	MGST3	microsomal glutathione S-transferase 3
317762	C14orf65	chromosome 14 open reading frame 65
5599	MAPK8	mitogen-activated protein kinase 8
55501	CHST12	carbohydrate (chondroitin 4) sulfotransferase 12
6004	RGS16	regulator of G-protein signalling 16
9400	RECQL5	RecQ protein-like 5
9055	PRC1	protein regulator of cytokinesis 1
22948	CCT5	chaperonin containing TCP1, subunit 5 (epsilon)
10081	PDCD7	programmed cell death 7
6853	SYN1	synapsin I
5097	PCDH1	protocadherin 1 (cadherin-like 1)
64786	TBC1D15	TBC1 domain family, member 15
2060	EPS15	epidermal growth factor receptor pathway substrate 15
57708	MIER1	mesoderm induction early response 1 homolog (Xenopus laevis)
8452	CUL3	cullin 3
25957	C6orf111	chromosome 6 open reading frame 111
354	KLK3	kallikrein-related peptidase 3
4817	NIT1	nitrilase 1
79017	C7orf24	chromosome 7 open reading frame 24
143282	C10orf13	chromosome 10 open reading frame 13
7402	UTRN	utrophin (homologous to dystrophin)
23059	CLUAP1	clusterin associated protein 1
57701	KIAA1602	KIAA1602
93621	MRFAP1	Mof4 family associated protein 1
3454	IFNAR1	interferon (alpha, beta and omega) receptor 1
51116	MRPS2	mitochondrial ribosomal protein S2
60559	SPCS3	signal peptidase complex subunit 3 homolog (S. cerevisiae)
7353	UFD1L	ubiquitin fusion degradation 1 like (yeast)
5066	PAM	peptidylglycine alpha-amidating monooxygenase

9925	ZBTB5	zinc finger and BTB domain containing 5
4915	NTRK2	neurotrophic tyrosine kinase, receptor, type 2
847	CAT	catalase
2982	GUCY1A3	guanylate cyclase 1, soluble, alpha 3
5997	RGS2	regulator of G-protein signalling 2, 24kDa
348235	FAM33A	family with sequence similarity 33, member A
10133	OPTN	optineurin
6596	SMARCA3	SWI/SNF related, matrix associated, actin dependent regulator of chromatin, subfamily a, member 3
51411	BIN2	bridging integrator 2
80325	ABTB1	ankyrin repeat and BTB (POZ) domain containing 1
80119	C15orf20	chromosome 15 open reading frame 20
5905	RANGAP1	Ran GTPase activating protein 1
30836	DNTTIP2	deoxynucleotidyltransferase, terminal, interacting protein 2
6638	SNRPN	small nuclear ribonucleoprotein polypeptide N
813	CALU	calumenin
64375	IKZF4	IKAROS family zinc finger 4 (Eos)
25875	LETMD1	LETM1 domain containing 1
54825	PCLKC	protocadherin LKC
6249	RSN	restin (Reed-Steinberg cell-expressed intermediate filament-associated protein)
54716	SLC6A20	solute carrier family 6 (proline IMINO transporter), member 20
91156	DKFZp434B1231	eEF1A2 binding protein
9246	UBE2L6	ubiquitin-conjugating enzyme E2L 6
54462	KIAA1128	KIAA1128
10539	TXNL2	thioredoxin-like 2
23150	FRMD4B	FERM domain containing 4B
9933	KIAA0020	KIAA0020
7257	TSNAX	translin-associated factor X
7187	TRAF3	TNF receptor-associated factor 3
57118	CAMK1D	calcium/calmodulin-dependent protein kinase ID
1479	CSTF3	cleavage stimulation factor, 3' pre-RNA, subunit 3, 77kDa
23034	SAMD4A	sterile alpha motif domain containing 4A
9639	ARHGEF10	Rho guanine nucleotide exchange factor (GEF) 10
84246	MED10	mediator of RNA polymerase II transcription, subunit 10 homolog (NUT2, S. cerevisiae)

3689	ITGB2	integrin, beta 2 (complement component 3 receptor 3 and 4 subunit)
65005	MRPL9	mitochondrial ribosomal protein L9
79758	DHRS12	dehydrogenase/reductase (SDR family) member 12
5630	PRPH	peripherin
80271	ITPKC	inositol 1,4,5-trisphosphate 3-kinase C
246243	RNASEH1	ribonuclease H1
5046	PCSK6	proprotein convertase subtilisin/kexin type 6
6286	S100P	S100 calcium binding protein P
50717	WDR42A	WD repeat domain 42A
10439	OLFM1	olfactomedin 1
10439	OLFM1	olfactomedin 1
55315	SLC29A3	solute carrier family 29 (nucleoside transporters), member 3
83986	ITFG3	integrin alpha FG-GAP repeat containing 3
23478	SEC11L1	SEC11-like 1 (S. cerevisiae)
171024	SYNPO2	synaptopodin 2
120863	DEPDC4	DEP domain containing 4
6464	SHC1	SHC (Src homology 2 domain containing) transforming protein 1
80790	CMIP	c-Maf-inducing protein
10982	MAPRE2	microtubule-associated protein, RP/EB family, member 2
399687	MYO18A	myosin XVIIIA
2041	EPHA1	EPH receptor A1
56852	RAD18	RAD18 homolog (S. cerevisiae)
6146	RPL22	ribosomal protein L22
3909	LAMA3	laminin, alpha 3
84173	RBED1	RNA binding motif and ELMO/CED-12 domain 1
7920	BAT5	HLA-B associated transcript 5
56886	UGCGL1	UDP-glucose ceramide glucosyltransferase-like 1
25983	NGDN	neuroguidin, EIF4E binding protein
8727	CTNNAL1	catenin (cadherin-associated protein), alpha-like 1
2530	FUT8	fucosyltransferase 8 (alpha (1,6) fucosyltransferase)
79573	TTC13	tetratricopeptide repeat domain 13
1843	DUSP1	dual specificity phosphatase 1
26011	ODZ4	odz, odd Oz/ten-m homolog 4 (Drosophila)
8829	NRP1	neuropilin 1
64333	ARHGAP9	Rho GTPase activating protein 9
9518	GDF15	growth differentiation factor 15
10557	RPP38	ribonuclease P/MRP 38kDa subunit
57455	REXO1	REX1, RNA exonuclease 1 homolog (S. cerevisiae)

339287	LOC339287	hypothetical protein LOC339287
9073	CLDN8	claudin 8
28512	NKIRAS1	NFKB inhibitor interacting Ras-like 1
54432	YIPF1	Yip1 domain family, member 1
84135	UTP15	UTP15, U3 small nucleolar ribonucleoprotein, homolog (S. cerevisiae)
51110	LACTB2	lactamase, beta 2
57605	PITPNM2	phosphatidylinositol transfer protein, membrane-associated 2
3030	HADHA	hydroxyacyl-Coenzyme A dehydrogenase/3-ketoacyl-Coenzyme A thiolase/enoyl-Coenzyme A hydratase (trifunctional protein), alpha subunit
9563	H6PD	hexose-6-phosphate dehydrogenase (glucose 1-dehydrogenase)
23316	CUTL2	cut-like 2 (Drosophila)
8987	GENX-3414	genethonin 1
8609	KLF7	Kruppel-like factor 7 (ubiquitous)
11164	NUDT5	nudix (nucleoside diphosphate linked moiety X)-type motif 5
10560	SLC19A2	solute carrier family 19 (thiamine transporter), member 2
79084	WDR77	WD repeat domain 77
8541	PPFIA3	protein tyrosine phosphatase, receptor type, f polypeptide (PTPRF), interacting protein (liprin), alpha 3
10324	KBTBD10	kelch repeat and BTB (POZ) domain containing 10
7840	ALMS1	Alstrom syndrome 1
25831	HECTD1	HECT domain containing 1
833	CARS	cysteinyl-tRNA synthetase
58487	ZF	HCF-binding transcription factor Zhangfei
9712	USP6NL	USP6 N-terminal like
26156	RSL1D1	ribosomal L1 domain containing 1
55769	ZNF83	zinc finger protein 83
25903	OLFML2B	olfactomedin-like 2B
6553	SLC9A5	solute carrier family 9 (sodium/hydrogen exchanger), member 5
2824	GPM6B	glycoprotein M6B
5264	PHYH	phytanoyl-CoA 2-hydroxylase
10418	SPON1	spondin 1, extracellular matrix protein
23081	JMJD2C	jumonji domain containing 2C
56882	CDC42SE1	CDC42 small effector 1
644	BLVRA	biliverdin reductase A
9486	CHST10	carbohydrate sulfotransferase 10
10762	NUP50	nucleoporin 50kDa
11221	DUSP10	dual specificity phosphatase 10

1731	SEPT1	septin 1
50489	CD207	CD207 molecule, langerin
30827	CXXC1	CXXC finger 1 (PHD domain)
8463	TEAD2	TEA domain family member 2
7038	TG	thyroglobulin
5034	P4HB	procollagen-proline, 2-oxoglutarate 4-dioxygenase (proline 4-hydroxylase), beta polypeptide
266812	NAP1L5	nucleosome assembly protein 1-like 5
6014	RIT2	Ras-like without CAAX 2
3291	HSD11B2	hydroxysteroid (11-beta) dehydrogenase 2
827	CAPN6	calpain 6
1837	DTNA	dystrobrevin, alpha
9223	MAGI1	membrane associated guanylate kinase, WW and PDZ domain containing 1
10464	C13orf24	chromosome 13 open reading frame 24
285704	RGMB	RGM domain family, member B
5993	RFX5	regulatory factor X, 5 (influences HLA class II expression)
64376	IKZF5	IKAROS family zinc finger 5 (Pegasus)
3710	ITPR3	inositol 1,4,5-triphosphate receptor, type 3
85450	KIAA1754	KIAA1754
10213	PSMD14	proteasome (prosome, macropain) 26S subunit, non-ATPase, 14
3553	IL1B	interleukin 1, beta
5684	PSMA3	proteasome (prosome, macropain) subunit, alpha type, 3
6738	TROVE2	TROVE domain family, member 2
25847	ANAPC13	anaphase promoting complex subunit 13
5074	PAWR	PRKC, apoptosis, WT1, regulator
9580	SOX13	SRY (sex determining region Y)-box 13
150572	SMYD1	SET and MYND domain containing 1
23061	TBC1D9B	TBC1 domain family, member 9B (with GRAM domain)
79188	TMEM43	transmembrane protein 43
51430	C1orf9	chromosome 1 open reading frame 9
23348	DOCK9	dedicator of cytokinesis 9
11164	NUDT5	nudix (nucleoside diphosphate linked moiety X)-type motif 5
23288	IQCE	IQ motif containing E
123	ADFP	adipose differentiation-related protein
1827	DSCR1	Down syndrome critical region gene 1
6732	SRPK1	SFRS protein kinase 1
3219	HOXB9	homeobox B9
60412	EXOC4	exocyst complex component 4
57680	CHD8	chromodomain helicase DNA binding protein 8

22802	CLCA4	chloride channel, calcium activated, family member 4
27248	C2orf30	chromosome 2 open reading frame 30
54987	C1orf123	chromosome 1 open reading frame 123
49855	ZNF291	zinc finger protein 291
80209	C13orf23	chromosome 13 open reading frame 23
26224	FBXL3	F-box and leucine-rich repeat protein 3
27243	CHMP2A	chromatin modifying protein 2A
10745	PHTF1	putative homeodomain transcription factor 1
4435	CITED1	Cbp/p300-interacting transactivator, with Glu/Asp-rich carboxy-terminal domain, 1
1880	EBI2	Epstein-Barr virus induced gene 2 (lymphocyte-specific G protein-coupled receptor)
26504	CNNM4	cyclin M4
2125	EVPL	envoplakin
6422	SFRP1	secreted frizzled-related protein 1
127544	IBRDC3	IBR domain containing 3
7071	KLF10	Kruppel-like factor 10
5469	PPARBP	PPAR binding protein
51703	ACSL5	acyl-CoA synthetase long-chain family member 5
57657	HCN3	hyperpolarization activated cyclic nucleotide-gated potassium channel 3
3939	LDHA	lactate dehydrogenase A
57482	KIAA1211	KIAA1211 protein
114882	OSBPL8	oxysterol binding protein-like 8
339287	LOC339287	hypothetical protein LOC339287
55520	ELAC1	elaC homolog 1 (E. coli)
64115	C10orf54	chromosome 10 open reading frame 54
408	ARRB1	arrestin, beta 1
93624	MGC21874	transcriptional adaptor 2 (ADA2 homolog, yeast)-beta
23670	TMEM2	transmembrane protein 2
11178	LZTS1	leucine zipper, putative tumor suppressor 1
221322	C6orf170	chromosome 6 open reading frame 170
203054	ADCK5	aarF domain containing kinase 5
10915	TCERG1	transcription elongation regulator 1
7175	TPR	translocated promoter region (to activated MET oncogene)
23154	NCDN	neurochondrin
2631	GBAS	glioblastoma amplified sequence
2078	ERG	v-ets erythroblastosis virus E26 oncogene homolog (avian)
150465	TTL	tubulin tyrosine ligase
84318	CCDC77	coiled-coil domain containing 77
56254	RNF20	ring finger protein 20
23095	KIF1B	kinesin family member 1B

23275	POFUT2	protein O-fucosyltransferase 2
9650	MTFR1	mitochondrial fission regulator 1
79767	ELMO3	engulfment and cell motility 3
6901	TAZ	tafazzin (cardiomyopathy, dilated 3A (X-linked); endocardial fibroelastosis 2; Barth syndrome)
57535	KIAA1324	KIAA1324
94056	SYAP1	synapse associated protein 1, SAP47 homolog (Drosophila)
471	ATIC	5-aminoimidazole-4-carboxamide ribonucleotide formyltransferase/IMP cyclohydrolase
57464	FAM40B	family with sequence similarity 40, member B
60625	DHX35	DEAH (Asp-Glu-Ala-His) box polypeptide 35
966	CD59	CD59 molecule, complement regulatory protein
2828	GPR4	G protein-coupled receptor 4
1386	ATF2	activating transcription factor 2
6813	STXBP2	syntaxin binding protein 2
1994	ELAVL1	ELAV (embryonic lethal, abnormal vision, Drosophila)-like 1 (Hu antigen R)
6484	ST3GAL4	ST3 beta-galactoside alpha-2,3-sialyltransferase 4
6122	RPL3	ribosomal protein L3
10902	BRD8	bromodomain containing 8
27018	NGFRAP1	nerve growth factor receptor (TNFRSF16) associated protein 1
34	ACADM	acyl-Coenzyme A dehydrogenase, C-4 to C-12 straight chain
7570	ZNF22	zinc finger protein 22 (KOX 15)
57685	CACHD1	cache domain containing 1
81567	TXNDC5	thioredoxin domain containing 5
8676	STX11	syntaxin 11
4292	MLH1	mutL homolog 1, colon cancer, nonpolyposis type 2 (E. coli)
10252	SPRY1	sprouty homolog 1, antagonist of FGF signaling (Drosophila)
9404	LPXN	leupaxin
10048	RANBP9	RAN binding protein 9
27352	RUTBC3	RUN and TBC1 domain containing 3
9673	SLC25A44	solute carrier family 25, member 44
23277	KIAA0664	KIAA0664
5051	PAFAH2	platelet-activating factor acetylhydrolase 2, 40kDa
2870	GRK6	G protein-coupled receptor kinase 6
55314	TMEM144	transmembrane protein 144
10714	POLD3	polymerase (DNA-directed), delta 3, accessory subunit

79009	DDX50	DEAD (Asp-Glu-Ala-Asp) box polypeptide 50
718	C3	complement component 3
9725	TMEM63A	transmembrane protein 63A
80144	FRAS1	Fraser syndrome 1
10947	AP3M2	adaptor-related protein complex 3, mu 2 subunit
51727	CMPK	cytidylate kinase
54940	OCIAD1	OCIA domain containing 1
50507	NOX4	NADPH oxidase 4
5002	SLC22A18	solute carrier family 22 (organic cation transporter), member 18
23122	CLASP2	cytoplasmic linker associated protein 2
5627	PROS1	protein S (alpha)
5204	PFDN5	prefoldin subunit 5
57216	VANGL2	vang-like 2 (van gogh, Drosophila)
9919	KIAA0310	KIAA0310
23085	ERC1	ELKS/RAB6-interacting/CAST family member 1
58155	PTBP2	polypyrimidine tract binding protein 2
4140	MARK3	MAP/microtubule affinity-regulating kinase 3
220972	MARCH8	membrane-associated ring finger (C3HC4) 8
9899	SV2B	synaptic vesicle glycoprotein 2B
8809	IL18R1	interleukin 18 receptor 1
9698	PUM1	pumilio homolog 1 (Drosophila)
54454	KIAA1240	KIAA1240 protein
80700	UBXD1	UBX domain containing 1
4739	NEDD9	neural precursor cell expressed, developmentally down-regulated 9
4145	MATK	megakaryocyte-associated tyrosine kinase
26052	DNM3	dynamin 3
7559	ZNF12	zinc finger protein 12
5522	PPP2R2C	protein phosphatase 2 (formerly 2A), regulatory subunit B (PR 52), gamma isoform
4054	LTBP3	latent transforming growth factor beta binding protein 3
4664	NAB1	NGFI-A binding protein 1 (EGR1 binding protein 1)
64856	VWA1	von Willebrand factor A domain containing 1
50848	F11R	F11 receptor
84254	CAMKK1	calcium/calmodulin-dependent protein kinase kinase 1, alpha
64342	HS1BP3	HCLS1 binding protein 3
6941	TCF19	transcription factor 19 (SC1)
55510	DDX43	DEAD (Asp-Glu-Ala-Asp) box polypeptide 43

27065	D4S234E	DNA segment on chromosome 4 (unique) 234 expressed sequence
1749	DLX5	distal-less homeobox 5
10492	SYNCRIP	synaptotagmin binding, cytoplasmic RNA interacting protein
51808	RNUXA	RNA U, small nuclear RNA export adaptor (phosphorylation regulated)
4045	LSAMP	limbic system-associated membrane protein
55677	IWS1	IWS1 homolog (S. cerevisiae)
3182	HNRPAB	heterogeneous nuclear ribonucleoprotein A/B
23515	MORC3	MORC family CW-type zinc finger 3
6421	SFPQ	splicing factor proline/glutamine-rich (polypyrimidine tract binding protein associated)
6770	STAR	steroidogenic acute regulator
80824	DUSP16	dual specificity phosphatase 16
53615	MBD3	methyl-CpG binding domain protein 3
54881	TEX10	testis expressed sequence 10
10494	STK25	serine/threonine kinase 25 (STE20 homolog, yeast)
10755	GIPC1	GIPC PDZ domain containing family, member 1
1439	CSF2RB	colony stimulating factor 2 receptor, beta, low-affinity (granulocyte-macrophage)
10237	SLC35B1	solute carrier family 35, member B1
158399	ZNF483	zinc finger protein 483
9093	DNAJA3	DnaJ (Hsp40) homolog, subfamily A, member 3
3836	KPNA1	karyopherin alpha 1 (importin alpha 5)
7039	TGFA	transforming growth factor, alpha
7039	TGFA	transforming growth factor, alpha
65010	SLC26A6	solute carrier family 26, member 6
389	RHOC	ras homolog gene family, member C
10286	BCAS2	breast carcinoma amplified sequence 2
64857	PLEKHG2	pleckstrin homology domain containing, family G (with RhoGef domain) member 2
3678	ITGA5	integrin, alpha 5 (fibronectin receptor, alpha polypeptide)
159	ADSS	adenylosuccinate synthase
4688	NCF2	neutrophil cytosolic factor 2 (65kDa, chronic granulomatous disease, autosomal 2)
64708	COPS7B	COP9 constitutive photomorphogenic homolog subunit 7B (Arabidopsis)
199692	ZNF627	zinc finger protein 627
23301	EHBP1	EH domain binding protein 1

3081	HGD	homogentisate 1,2-dioxygenase (homogentisate oxidase)
1910	EDNRB	endothelin receptor type B
54622	ARL15	ADP-ribosylation factor-like 15
7373	COL14A1	collagen, type XIV, alpha 1 (undulin)
57488	FAM62B	family with sequence similarity 62 (C2 domain containing) member B
9950	GOLGA5	golgi autoantigen, golgin subfamily a, 5
55075	UACA	uveal autoantigen with coiled-coil domains and ankyrin repeats
6125	RPL5	ribosomal protein L5
1594	CYP27B1	cytochrome P450, family 27, subfamily B, polypeptide 1
221178	SPATA13	spermatogenesis associated 13
84823	LMNB2	lamin B2
23552	CCRK	cell cycle related kinase
2354	FOSB	FBJ murine osteosarcoma viral oncogene homolog B
9669	EIF5B	eukaryotic translation initiation factor 5B
9063	PIAS2	protein inhibitor of activated STAT, 2
1956	EGFR	epidermal growth factor receptor (erythroblastic leukemia viral (v-erb-b) oncogene homolog, avian)
26128	KIAA1279	KIAA1279
10131	TRAP1	TNF receptor-associated protein 1
50717	WDR42A	WD repeat domain 42A
55589	BMP2K	BMP2 inducible kinase
9927	MFN2	mitofusin 2
9927	MFN2	mitofusin 2
8218	CLTCL1	clathrin, heavy chain-like 1
8218	CLTCL1	clathrin, heavy chain-like 1
6242	RTKN	rhotekin
10422	UBADC1	ubiquitin associated domain containing 1
7572	ZNF24	zinc finger protein 24
22837	COBLL1	COBL-like 1
9453	GGPS1	geranylgeranyl diphosphate synthase 1
6326	SCN2A2	sodium channel, voltage-gated, type II, alpha 2
4312	MMP1	matrix metallopeptidase 1 (interstitial collagenase)
10664	CTCF	CCCTC-binding factor (zinc finger protein)
8910	SGCE	sarcoglycan, epsilon
10121	ACTR1A	ARP1 actin-related protein 1 homolog A, centractin alpha (yeast)
84900	TMEM118	transmembrane protein 118
57460	PPM1H	protein phosphatase 1H (PP2C domain containing)
6671	SP4	Sp4 transcription factor

9765	ZFYVE16	zinc finger, FYVE domain containing 16
79656	C1orf165	chromosome 1 open reading frame 165
50488	MINK1	misshapen-like kinase 1 (zebrafish)
57826	RAP2C	RAP2C, member of RAS oncogene family
23412	COMMD3	COMM domain containing 3
3632	INPP5A	inositol polyphosphate-5-phosphatase, 40kDa
23213	SULF1	sulfatase 1
5571	PRKAG1	protein kinase, AMP-activated, gamma 1 non-catalytic subunit
5653	KLK6	kallikrein-related peptidase 6
1192	CLIC1	chloride intracellular channel 1
51582	AZIN1	antizyme inhibitor 1
171023	ASXL1	additional sex combs like 1 (Drosophila)
5495	PPM1B	protein phosphatase 1B (formerly 2C), magnesium-dependent, beta isoform
55917	CTTNBP2NL	CTTNBP2 N-terminal like
55917	CTTNBP2NL	CTTNBP2 N-terminal like
9732	DOCK4	dedicator of cytokinesis 4
25938	C14orf125	chromosome 14 open reading frame 125
8895	CPNE3	copine III
57700	KIAA1600	KIAA1600
114881	OSBPL7	oxysterol binding protein-like 7
64122	FN3K	fructosamine 3 kinase
84186	ZCCHC7	zinc finger, CCHC domain containing 7
1374	CPT1A	carnitine palmitoyltransferase 1A (liver)
65983	GRAMD3	GRAM domain containing 3
5129	PCTK3	PCTAIRE protein kinase 3
56910	STARD7	START domain containing 7
5864	RAB3A	RAB3A, member RAS oncogene family
57549	IGSF9	immunoglobulin superfamily, member 9
11067	C10orf10	chromosome 10 open reading frame 10
2901	GRIK5	glutamate receptor, ionotropic, kainate 5
27340	UTP20	UTP20, small subunit (SSU) processome component, homolog (yeast)
6821	SUOX	sulfite oxidase
10568	SLC34A2	solute carrier family 34 (sodium phosphate), member 2
112483	SAT2	spermidine/spermine N1-acetyltransferase 2
6434	SFRS10	splicing factor, arginine/serine-rich 10 (transformer 2 homolog, Drosophila)
4552	MTRR	5-methyltetrahydrofolate-homocysteine methyltransferase reductase
56995	TULP4	tubby like protein 4

6809	STX3	syntaxin 3
23228	PLCL2	phospholipase C-like 2
8560	DEGS1	degenerative spermatocyte homolog 1, lipid desaturase (Drosophila)
27095	TRAPPC3	trafficking protein particle complex 3
25813	SAMM50	sorting and assembly machinery component 50 homolog (S. cerevisiae)
8888	MCM3AP	MCM3 minichromosome maintenance deficient 3 (S. cerevisiae) associated protein
4283	CXCL9	chemokine (C-X-C motif) ligand 9
92454	PRR8	proline rich 8
57631	LRCH2	leucine-rich repeats and calponin homology (CH) domain containing 2
60681	FKBP10	FK506 binding protein 10, 65 kDa
4199	ME1	malic enzyme 1, NADP(+)-dependent, cytosolic
4199	ME1	malic enzyme 1, NADP(+)-dependent, cytosolic
8554	PIAS1	protein inhibitor of activated STAT, 1
57162	PELI1	pellino homolog 1 (Drosophila)
2113	ETS1	v-ets erythroblastosis virus E26 oncogene homolog 1 (avian)
51094	ADIPOR1	adiponectin receptor 1
23317	DNAJC13	DnaJ (Hsp40) homolog, subfamily C, member 13
579	BAPX1	bagpipe homeobox homolog 1 (Drosophila)
404093	CUEDC1	CUE domain containing 1
9980	DOPEY2	dopey family member 2
23007	PLCH1	phospholipase C, eta 1
2686	GGTL3	gamma-glutamyltransferase-like 3
83892	KCTD10	potassium channel tetramerisation domain containing 10
10989	IMMT	inner membrane protein, mitochondrial (mitofilin)
128	ADH5	alcohol dehydrogenase 5 (class III), chi polypeptide
7043	TGFB3	transforming growth factor, beta 3
2145	EZH1	enhancer of zeste homolog 1 (Drosophila)
29123	ANKRD11	ankyrin repeat domain 11
11001	SLC27A2	solute carrier family 27 (fatty acid transporter), member 2
547	KIF1A	kinesin family member 1A
94241	TP53INP1	tumor protein p53 inducible nuclear protein 1
23463	ICMT	isoprenylcysteine carboxyl methyltransferase
81555	YIPF5	Yip1 domain family, member 5
79078	C1orf50	chromosome 1 open reading frame 50

4839	NOL1	nucleolar protein 1, 120kDa
7347	UCHL3	ubiquitin carboxyl-terminal esterase L3 (ubiquitin thiolesterase)
26301	GBGT1	globoside alpha-1,3-N-acetylgalactosaminyltransferase 1
1676	DFFA	DNA fragmentation factor, 45kDa, alpha polypeptide
54617	INOC1	INO80 complex homolog 1 (S. cerevisiae)
51673	CGI-38	brain specific protein
6625	SNRP70	small nuclear ribonucleoprotein 70kDa polypeptide (RNP antigen)
64223	GBL	G protein beta subunit-like
199745	THAP8	THAP domain containing 8
79876	UBE1DC1	ubiquitin-activating enzyme E1-domain containing 1
4072	TACSTD1	tumor-associated calcium signal transducer 1
11197	WIF1	WNT inhibitory factor 1
26585	GREM1	gremlin 1, cysteine knot superfamily, homolog (Xenopus laevis)
6204	RPS10	ribosomal protein S10
4023	LPL	lipoprotein lipase
10635	RAD51AP1	RAD51 associated protein 1
2926	GRSF1	G-rich RNA sequence binding factor 1
56997	CABC1	chaperone, ABC1 activity of bc1 complex homolog (S. pombe)
815	CAMK2A	calcium/calmodulin-dependent protein kinase (CaM kinase) II alpha
23366	KIAA0895	KIAA0895 protein
10979	PLEKHC1	pleckstrin homology domain containing, family C (with FERM domain) member 1
1501	CTNND2	catenin (cadherin-associated protein), delta 2 (neural plakophilin-related arm-repeat protein)
820	CAMP	cathelicidin antimicrobial peptide
9727	RAB11FIP3	RAB11 family interacting protein 3 (class II)
55743	CHFR	checkpoint with forkhead and ring finger domains
84437	KIAA1826	KIAA1826
1272	CNTN1	contactin 1
26115	TANC2	tetratricopeptide repeat, ankyrin repeat and coiled-coil containing 2
55230	USP40	ubiquitin specific peptidase 40
23405	DICER1	Dicer1, Dcr-1 homolog (Drosophila)
1607	DGKB	diacylglycerol kinase, beta 90kDa
55701	FLJ10357	hypothetical protein FLJ10357
108	ADCY2	adenylate cyclase 2 (brain)
5650	KLK7	kallikrein-related peptidase 7

9685	CLINT1	clathrin interactor 1
152137	CCDC50	coiled-coil domain containing 50
3394	IRF8	interferon regulatory factor 8
857	CAV1	caveolin 1, caveolae protein, 22kDa
8496	PPFIBP1	PTPRF interacting protein, binding protein 1 (liprin beta 1)
4791	NFKB2	nuclear factor of kappa light polypeptide gene enhancer in B-cells 2 (p49/p100)
25948	KBTBD2	kelch repeat and BTB (POZ) domain containing 2
1153	CIRBP	cold inducible RNA binding protein
2219	FCN1	ficolin (collagen/fibrinogen domain containing) 1
1615	DARS	aspartyl-tRNA synthetase
55624	POMGNT1	protein O-linked mannose beta1,2-N-acetylglucosaminyltransferase
2057	EPOR	erythropoietin receptor
23435	TARDBP	TAR DNA binding protein
23435	TARDBP	TAR DNA binding protein
7022	TFAP2C	transcription factor AP-2 gamma (activating enhancer binding protein 2 gamma)
9354	UBE4A	ubiquitination factor E4A (UFD2 homolog, yeast)
2213	FCGR2B	Fc fragment of IgG, low affinity IIb, receptor (CD32)
92241	RCSD1	RCSD domain containing 1
2588	GALNS	galactosamine (N-acetyl)-6-sulfate sulfatase (Morquio syndrome, mucopolysaccharidosis type IVA)
10099	TSPAN3	tetraspanin 3
60686	C14orf93	chromosome 14 open reading frame 93
4846	NOS3	nitric oxide synthase 3 (endothelial cell)
79086	C19orf42	chromosome 19 open reading frame 42
79802	KIAA1822L	KIAA1822-like
57553	MICAL3	microtubule associated monoxygenase, calponin and LIM domain containing 3
9551	ATP5J2	ATP synthase, H+ transporting, mitochondrial F0 complex, subunit F2
2934	GSN	gelsolin (amyloidosis, Finnish type)
8939	FUBP3	far upstream element (FUSE) binding protein 3
6405	SEMA3F	sema domain, immunoglobulin domain (Ig), short basic domain, secreted, (semaphorin) 3F
719	C3AR1	complement component 3a receptor 1
55118	CRTAC1	cartilage acidic protein 1
6608	SMO	smoothened homolog (Drosophila)

9903	KLHL21	kelch-like 21 (Drosophila)
23389	THRAP2	thyroid hormone receptor associated protein 2
7533	YWHAH	tyrosine 3-monooxygenase/tryptophan 5-monooxygenase activation protein, eta polypeptide
25999	CLIPR-59	CLIP-170-related protein
55224	ETNK2	ethanolamine kinase 2
400	ARL1	ADP-ribosylation factor-like 1
157247	MGC27345	hypothetical protein MGC27345
54838	C10orf26	chromosome 10 open reading frame 26
10806	SDCCAG8	serologically defined colon cancer antigen 8
3667	IRS1	insulin receptor substrate 1
10171	RCL1	RNA terminal phosphate cyclase-like 1
863	CBFA2T3	core-binding factor, runt domain, alpha subunit 2; translocated to, 3
5338	PLD2	phospholipase D2
9138	ARHGEF1	Rho guanine nucleotide exchange factor (GEF) 1
1213	CLTC	clathrin, heavy chain (Hc)
55215	KIAA1794	KIAA1794
3658	IREB2	iron-responsive element binding protein 2
1908	EDN3	endothelin 3
81671	TMEM49	transmembrane protein 49
284459	HKR1	GLI-Kruppel family member HKR1
115209	OMA1	OMA1 homolog, zinc metallopeptidase (S. cerevisiae)
5184	PEPD	peptidase D
5935	RBM3	RNA binding motif (RNP1, RRM) protein 3
55081	IFT57	intraflagellar transport 57 homolog (Chlamydomonas)
9658	ZNF516	zinc finger protein 516
1140	CHRNB1	cholinergic receptor, nicotinic, beta 1 (muscle)
6453	ITSN1	intersectin 1 (SH3 domain protein)
8559	PRPF18	PRP18 pre-mRNA processing factor 18 homolog (S. cerevisiae)
51340	CRNKL1	Crn, crooked neck-like 1 (Drosophila)
6867	TACC1	transforming, acidic coiled-coil containing protein 1
7586	ZKSCAN1	zinc finger with KRAB and SCAN domains 1
2139	EYA2	eyes absent homolog 2 (Drosophila)
10891	PPARGC1A	peroxisome proliferator-activated receptor gamma, coactivator 1 alpha
8318	CDC45L	CDC45 cell division cycle 45-like (S. cerevisiae)

8318	CDC45L	CDC45 cell division cycle 45-like (S. cerevisiae)
29841	GRHL1	grainyhead-like 1 (Drosophila)
677	ZFP36L1	zinc finger protein 36, C3H type-like 1
57606	SLAIN2	SLAIN motif family, member 2
51704	GPRC5B	G protein-coupled receptor, family C, group 5, member B
84986	ARHGAP19	Rho GTPase activating protein 19
6356	CCL11	chemokine (C-C motif) ligand 11
201134	CCDC46	coiled-coil domain containing 46
10797	MTHFD2	methylenetetrahydrofolate dehydrogenase (NADP+ dependent) 2, methenyltetrahydrofolate cyclohydrolase
8027	STAM	signal transducing adaptor molecule (SH3 domain and ITAM motif) 1
91419	XRCC6BP1	XRCC6 binding protein 1
4846	NOS3	nitric oxide synthase 3 (endothelial cell)
10914	PAPOLA	poly(A) polymerase alpha
51093	C1orf66	chromosome 1 open reading frame 66
3655	ITGA6	integrin, alpha 6
1382	CRABP2	cellular retinoic acid binding protein 2
2214	FCGR3A	Fc fragment of IgG, low affinity IIIa, receptor (CD16a)
11218	DDX20	DEAD (Asp-Glu-Ala-Asp) box polypeptide 20
81563	C1orf21	chromosome 1 open reading frame 21
6641	SNTB1	syntrophin, beta 1 (dystrophin-associated protein A1, 59kDa, basic component 1)
55716	LMBR1L	limb region 1 homolog (mouse)-like
9044	BTAF1	BTAF1 RNA polymerase II, B-TFIID transcription factor-associated, 170kDa (Mot1 homolog, S. cerevisiae)
5033	P4HA1	procollagen-proline, 2-oxoglutarate 4-dioxygenase (proline 4-hydroxylase), alpha polypeptide I
51155	HN1	hematological and neurological expressed 1
146923	RUNDC1	RUN domain containing 1
23127	GLT25D2	glycosyltransferase 25 domain containing 2
5360	PLTP	phospholipid transfer protein
10102	TSFM	Ts translation elongation factor, mitochondrial
7741	ZNF187	zinc finger protein 187
5709	PSMD3	proteasome (prosome, macropain) 26S subunit, non-ATPase, 3
351	APP	amyloid beta (A4) precursor protein (peptidase nexin-II, Alzheimer disease)

57575	PCDH10	protocadherin 10
8503	PIK3R3	phosphoinositide-3-kinase, regulatory subunit 3 (p55, gamma)
6376	CX3CL1	chemokine (C-X3-C motif) ligand 1
1031	CDKN2C	cyclin-dependent kinase inhibitor 2C (p18, inhibits CDK4)
81607	PVRL4	poliovirus receptor-related 4
6171	RPL41	ribosomal protein L41
9840	KIAA0748	KIAA0748
5354	PLP1	proteolipid protein 1 (Pelizaeus-Merzbacher disease, spastic paraplegia 2, uncomplicated)
5007	OSBP	oxysterol binding protein
55719	C10orf6	chromosome 10 open reading frame 6
57414	RHBDD2	rhomboid domain containing 2
55614	C20orf23	chromosome 20 open reading frame 23
10098	TSPAN5	tetraspanin 5
699	BUB1	BUB1 budding uninhibited by benzimidazoles 1 homolog (yeast)
55612	C20orf42	chromosome 20 open reading frame 42
10632	ATP5L	ATP synthase, H+ transporting, mitochondrial F0 complex, subunit G
55858	TMEM165	transmembrane protein 165
3815	KIT	v-kit Hardy-Zuckerman 4 feline sarcoma viral oncogene homolog
29890	RBM15B	RNA binding motif protein 15B
83543	C9orf58	chromosome 9 open reading frame 58
1036	CDO1	cysteine dioxygenase, type I
57326	PBXIP1	pre-B-cell leukemia transcription factor interacting protein 1
84059	GPR98	G protein-coupled receptor 98
22894	KIAA1008	KIAA1008
10174	SORBS3	sorbin and SH3 domain containing 3
10174	SORBS3	sorbin and SH3 domain containing 3
135112	NCOA7	nuclear receptor coactivator 7
27236	ARFIP1	ADP-ribosylation factor interacting protein 1 (arfaptin 1)
10607	TBL3	transducin (beta)-like 3
55727	BTBD7	BTB (POZ) domain containing 7
22881	ANKRD6	ankyrin repeat domain 6
79718	TBL1XR1	transducin (beta)-like 1X-linked receptor 1
91	ACVR1B	activin A receptor, type IB
2568	GABRP	gamma-aminobutyric acid (GABA) A receptor, pi
714	C1QC	complement component 1, q subcomponent, C chain
761	CA3	carbonic anhydrase III, muscle specific
92715	WDR85	WD repeat domain 85
79832	QSER1	glutamine and serine rich 1

10144	FAM13A1	family with sequence similarity 13, member A1
83719	YPEL3	yippee-like 3 (Drosophila)
1522	CTSZ	cathepsin Z
51373	MRPS17	mitochondrial ribosomal protein S17
4846	NOS3	nitric oxide synthase 3 (endothelial cell)
590	BCHE	butyrylcholinesterase
84498	FAM120B	family with sequence similarity 120B
79654	HECTD3	HECT domain containing 3
6498	SKIL	SKI-like
10476	ATP5H	ATP synthase, H+ transporting, mitochondrial F0 complex, subunit d
51246	SCOTIN	scotin
4940	OAS3	2'-5'-oligoadenylate synthetase 3, 100kDa
55713	ZNF334	zinc finger protein 334
8476	CDC42BPA	CDC42 binding protein kinase alpha (DMPK-like)
80023	NRSN2	neurensin 2
10653	SPINT2	serine peptidase inhibitor, Kunitz type, 2
171568	POLR3H	polymerase (RNA) III (DNA directed) polypeptide H (22.9kD)
79939	SLC35E1	solute carrier family 35, member E1
79648	MCPH1	microcephaly, primary autosomal recessive 1
81846	SBF2	SET binding factor 2
7169	TPM2	tropomyosin 2 (beta)
26287	ANKRD2	ankyrin repeat domain 2 (stretch responsive muscle)
10966	RAB40B	RAB40B, member RAS oncogene family
7113	TMPRSS2	transmembrane protease, serine 2
7321	UBE2D1	ubiquitin-conjugating enzyme E2D 1 (UBC4/5 homolog, yeast)
23623	RUSC1	RUN and SH3 domain containing 1
5920	RARRES3	retinoic acid receptor responder (tazarotene induced) 3
5523	PPP2R3A	protein phosphatase 2 (formerly 2A), regulatory subunit B'', alpha
55709	KBTBD4	kelch repeat and BTB (POZ) domain containing 4
57062	DDX24	DEAD (Asp-Glu-Ala-Asp) box polypeptide 24
6662	SOX9	SRY (sex determining region Y)-box 9 (campomelic dysplasia, autosomal sex-reversal)
9985	REC8L1	REC8-like 1 (yeast)
1308	COL17A1	collagen, type XVII, alpha 1
51136	LOC51136	PTD016 protein

27238	GPKOW	G patch domain and KOW motifs
1300	COL10A1	collagen, type X, alpha 1(Schmid metaphyseal chondrodysplasia)
