# Supplementary material for: Evidence that molecular changes in cells occur before morphological alterations during the progression of breast ductal carcinoma
Source: Breast Cancer Res. 2008 Oct 17;10(5):R87. doi: 10.1186/bcr2157 (PMC2614523; doi:10.1186/bcr2157)
Supplement: Additional file 2 — Differentially expressed genes: pure DCIS, in situ component of DCIS-IDC, and IDC lesions. Presented is a table listing the differentially expressed genes among tumor cells from pure DCIS, in situ component of DCIS-IDC, and IDC lesions. [file bcr2157-S2.pdf]

Additional Data File 2 - 90 genes

Comparison among pure DCIS, *in situ* component of DCIS-IDC and IDC

GeneID	Symbol	Description
51061	TXNDC11	thioredoxin domain containing 11
29965	C16orf5	chromosome 16 open reading frame 5
51093	C1orf66	chromosome 1 open reading frame 66
23154	NCDN	neurochondrin
1140	CHRNB1	cholinergic receptor, beta 1 (muscle), protein-coding
9486	CHST10	carbohydrate sulfotransferase 10
4140	MARK3	MAP/microtubule affinity-regulating kinase 3
114881	OSBPL7	oxysterol binding protein-like 7
55917	CTTNBP2NL	CTTNBP2 N-terminal like
93624	MGC21874	transcriptional adaptor 2 (ADA2 homolog) protein-coding
53615	MBD3	methyl-CpG binding domain protein 3
3394	IRF8	interferon regulatory factor 8
57605	PITPNM2	phosphatidylinositol transfer protein, protein-coding
55612	C20orf42	chromosome 20 open reading frame 42
10539	TXNL2	thioredoxin-like 2
23203	PMPCA	peptidase (mitochondrial processing) alpha
23034	SAMD4A	sterile alpha motif domain containing 4A
26301	GBGT1	globoside alpha-1, protein-coding
54838	C10orf26	chromosome 10 open reading frame 26
6122	RPL3	ribosomal protein L3
23085	ERC1	ELKS/RAB6-interacting/CAST family member 1
7840	ALMS1	Alstrom syndrome 1
2219	FCN1	ficolin (collagen/fibrinogen domain containing) 1
5184	PEPD	peptidase D
9580	SOX13	SRY (sex determining region Y)-box 13
54716	SLC6A20	solute carrier family 6 (proline IMINO transporter), protein-coding
10131	TRAP1	TNF receptor-associated protein 1
23277	KIAA0664	KIAA0664
9985	REC8L1	REC8-like 1 (yeast)
9404	LPXN	leupaxin
8676	STX11	syntaxin 11
27340	UTP20	UTP20, homolog (yeast), protein-coding
60625	DHX35	DEAH (Asp-Glu-Ala-His) box polypeptide 35
57575	PCDH10	protocadherin 10
2057	EPOR	erythropoietin receptor
160760	PPTC7	PTC7 protein phosphatase homolog (S. cerevisiae)
8318	CDC45L	CDC45 cell division cycle 45-like (S. cerevisiae)
123	ADFP	adipose differentiation-related protein
57464	FAM40B	family with sequence similarity 40, protein-coding
54825	PCLKC	protocadherin LKC
4054	LTBP3	latent transforming growth factor beta binding protein 3
10755	GIPC1	GIPC PDZ domain containing family, protein-coding
10714	POLD3	polymerase (DNA-directed), accessory subunit, protein-coding

1300	COL10A1	collagen, type X, alpha 1(Schmid metaphyseal chondrodysplasia)
10464	C13orf24	chromosome 13 open reading frame 24
79758	DHRS12	dehydrogenase/reductase (SDR family) member 12
9840	KIAA0748	KIAA0748
8895	CPNE3	copine III
25875	LETMD1	LETM1 domain containing 1
6553	SLC9A5	solute carrier family 9 (sodium/hydrogen exchanger),protein-coding
285704	RGMB	RGM domain family, protein-coding
51373	MRPS17	mitochondrial ribosomal protein S17
2041	EPHA1	EPH receptor A1
54617	INOC1	INO80 complex homolog 1 (S. cerevisiae)
25999	CLIPR-59	CLIP-170-related protein
8463	TEAD2	TEA domain family member 2
9732	DOCK4	dedicator of cytokinesis 4
10607	TBL3	transducin (beta)-like 3
8939	FUBP3	far upstream element (FUSE) binding protein 3
203054	ADCK5	aarF domain containing kinase 5
57482	KIAA1211	KIAA1211 protein
10745	PHTF1	putative homeodomain transcription factor 1
81555	YIPF5	Yip1 domain family, protein-coding
3632	INPP5A	inositol polyphosphate-5-phosphatase, protein-coding
58533	SNX6	sorting nexin 6
80271	ITPKC	inositol 1, 5-trisphosphate 3-kinase C, protein-coding
10982	MAPRE2	microtubule-associated protein, member 2, protein-coding
6464	SHC1	SHC (Src homology 2 domain containing) transforming protein 1
81563	C1orf21	chromosome 1 open reading frame 21
6625	SNRP70	small nuclear ribonucleoprotein 70kDa polypeptide (RNP antigen)
108	ADCY2	adenylate cyclase 2 (brain)
79009	DDX50	DEAD (Asp-Glu-Ala-Asp) box polypeptide 50
80209	C13orf23	chromosome 13 open reading frame 23
4739	NEDD9	neural precursor cell expressed, protein-coding
25831	HECTD1	HECT domain containing 1
57631	LRCH2	leucine-rich repeats and calponin homology (CH) domain containing 2
84246	MED10	mediator of RNA polymerase II transcription, S. cerevisiae, protein-coding
10244	RABEPK	Rab9 effector protein with kelch motifs
3101	HK3	hexokinase 3 (white cell)
9570	GOSR2	golgi SNAP receptor complex member 2
25804	LSM4	LSM4 homolog, U6 small nuclear RNA associated (S. cerevisiae)
9334	B4GALT5	UDP-Gal:betaGlcNAc beta 1,4- galactosyltransferase, polypeptide 5
5322	PLA2G5	phospholipase A2, group V
8945	BTRC	beta-transducin repeat containing

22898	DENND3	KIAA0870 protein
563	AZGP1	AZGP1- alpha-2-glycoprotein 1
6117	RPA1	replication protein A1, 70kDa
5865	RAB3B	RAB3B, member RAS oncogene family
55731	C17orf63	chromosome 17 open reading frame 63
