# Supplementary material for: Evidence that molecular changes in cells occur before morphological alterations during the progression of breast ductal carcinoma
Source: Breast Cancer Res. 2008 Oct 17;10(5):R87. doi: 10.1186/bcr2157 (PMC2614523; doi:10.1186/bcr2157)
Supplement: Additional file 3 — Differentially expressed genes: non-neoplastic, pure DCIS, and in situ component of DCIS-IDC lesions. Presented is a table listing the differentially expressed genes among cells from non-neoplastic, pure DCIS, and in situ component of DCIS-IDC lesions. [file bcr2157-S3.pdf]

Additional Data File 3 - 785 genes

Comparison among Non-neoplastic cells, pure DCIS, *in situ* component of DCIS-IDC

GeneID	Symbol	Description
9337	CNOT8	CCR4-NOT transcription complex, subunit 8
51393	TRPV2	transient receptor potential cation channel, subfamily V, member 2
55893	ZNF395	zinc finger protein 395
23048	FNBP1	formin binding protein 1
23048	FNBP1	formin binding protein 1
55777	MBD5	methyl-CpG binding domain protein 5
23451	SF3B1	splicing factor 3b, subunit 1, 155kDa
55256	ADI1	acireductone dioxygenase 1
79157	ET	hypothetical protein ET
4784	NFIX	nuclear factor I/X (CCAAT-binding transcription factor)
114112	TXNRD3	thioredoxin reductase 3
9055	PRC1	protein regulator of cytokinesis 1
9325	TRIP4	thyroid hormone receptor interactor 4
6638	SNRPN	small nuclear ribonucleoprotein polypeptide N
6249	RSN	restin (Reed-Steinberg cell-expressed intermediate filament-associated protein)
5630	PRPH	peripherin
5046	PCSK6	proprotein convertase subtilisin/kexin type 6
57455	REXO1	REX1, RNA exonuclease 1 homolog (S. cerevisiae)
54432	YIPF1	Yip1 domain family, member 1
84135	UTP15	UTP15, U3 small nucleolar ribonucleoprotein, homolog (S. cerevisiae)
3030	HADHA	hydroxyacyl-Coenzyme A dehydrogenase/3-ketoacyl-Coenzyme A thiolase/enoyl-Coenzyme A hydratase (trifunctional protein), alpha subunit
9563	H6PD	hexose-6-phosphate dehydrogenase (glucose 1-dehydrogenase)
9989	PPP4R1	protein phosphatase 4, regulatory subunit 1
154807	VKORC1L1	vitamin K epoxide reductase complex, subunit 1-like 1
54876	C4orf30	chromosome 4 open reading frame 30
5603	MAPK13	mitogen-activated protein kinase 13
3710	ITPR3	inositol 1,4,5-triphosphate receptor, type 3
10213	PSMD14	proteasome (prosome, macropain) 26S subunit, non-ATPase, 14
51430	C1orf9	chromosome 1 open reading frame 9
7884	SLBP	stem-loop (histone) binding protein
57231	SNX14	sorting nexin 14
7006	TEC	tec protein tyrosine kinase
64645	HIAT1	hippocampus abundant transcript 1

9920	KBTBD11	kelch repeat and BTB (POZ) domain containing 11
9650	MTFR1	mitochondrial fission regulator 1
57535	KIAA1324	KIAA1324
34	ACADM	acyl-Coenzyme A dehydrogenase, C-4 to C-12 straight chain
84769	FKSG24	hypothetical protein MGC12972
2870	GRK6	G protein-coupled receptor kinase 6
2768	GNA12	guanine nucleotide binding protein (G protein) alpha 12
10947	AP3M2	adaptor-related protein complex 3, mu 2 subunit
50848	F11R	F11 receptor
6941	TCF19	transcription factor 19 (SC1)
6770	STAR	steroidogenic acute regulator
1439	CSF2RB	colony stimulating factor 2 receptor, beta, low-affinity (granulocyte-macrophage)
3836	KPNA1	karyopherin alpha 1 (importin alpha 5)
159	ADSS	adenylosuccinate synthase
10269	ZMPSTE24	zinc metallopeptidase (STE24 homolog, yeast)
2889	RAPGEF1	Rap guanine nucleotide exchange factor (GEF) 1
9950	GOLGA5	golgi autoantigen, golgin subfamily a, 5
1594	CYP27B1	cytochrome P450, family 27, subfamily B, polypeptide 1
84823	LMNB2	lamin B2
26128	KIAA1279	KIAA1279
7572	ZNF24	zinc finger protein 24
9453	GGPS1	geranylgeranyl diphosphate synthase 1
4312	MMP1	matrix metallopeptidase 1 (interstitial collagenase)
9706	ULK2	unc-51-like kinase 2 (C. elegans)
400673	VMAC	vimentin-type IF-associated coiled-coil protein
5571	PRKAG1	protein kinase, AMP-activated, gamma 1 non-catalytic subunit
51582	AZIN1	antizyme inhibitor 1
57700	KIAA1600	KIAA1600
56910	STARD7	START domain containing 7
10964	IFI44L	interferon-induced protein 44-like
23228	PLCL2	phospholipase C-like 2
8888	MCM3AP	MCM3 minichromosome maintenance deficient 3 (S. cerevisiae) associated protein
8554	PIAS1	protein inhibitor of activated STAT, 1
23317	DNAJC13	DnaJ (Hsp40) homolog, subfamily C, member 13
29123	ANKRD11	ankyrin repeat domain 11
84502	JPH4	junctophilin 4

7347	UCHL3	ubiquitin carboxyl-terminal esterase L3 (ubiquitin thiolesterase)
10848	PPP1R13L	protein phosphatase 1, regulatory (inhibitor) subunit 13 like
7508	XPC	xeroderma pigmentosum, complementation group C
51673	CGI-38	brain specific protein
199745	THAP8	THAP domain containing 8
26585	GREM1	gremlin 1, cysteine knot superfamily, homolog (Xenopus laevis)
8741	TNFSF13	tumor necrosis factor (ligand) superfamily, member 13
815	CAMK2A	calcium/calmodulin-dependent protein kinase (CaM kinase) II alpha
84437	KIAA1826	KIAA1826
8496	PPFIBP1	PTPRF interacting protein, binding protein 1 (liprin beta 1)
4791	NFKB2	nuclear factor of kappa light polypeptide gene enhancer in B-cells 2 (p49/p100)
9354	UBE4A	ubiquitination factor E4A (UFD2 homolog, yeast)
23389	THRAP2	thyroid hormone receptor associated protein 2
55107	TMEM16A	transmembrane protein 16A
5338	PLD2	phospholipase D2
10891	PPARGC1A	peroxisome proliferator-activated receptor gamma, coactivator 1 alpha
91419	XRCC6BP1	XRCC6 binding protein 1
83604	TMEM47	transmembrane protein 47
8862	APLN	apelin, AGTRL1 ligand
55716	LMBR1L	limb region 1 homolog (mouse)-like
23127	GLT25D2	glycosyltransferase 25 domain containing 2
4801	NFYB	nuclear transcription factor Y, beta
1031	CDKN2C	cyclin-dependent kinase inhibitor 2C (p18, inhibits CDK4)
5007	OSBP	oxysterol binding protein
360	AQP3	aquaporin 3 (Gill blood group)
22894	KIAA1008	KIAA1008
3964	LGALS8	lectin, galactoside-binding, soluble, 8 (galectin 8)
4036	LRP2	low density lipoprotein-related protein 2
79145	CHCHD7	coiled-coil-helix-coiled-coil-helix domain containing 7
10144	FAM13A1	family with sequence similarity 13, member A1
90410	IFT20	intraflagellar transport 20 homolog (Chlamydomonas)
8476	CDC42BPA	CDC42 binding protein kinase alpha (DMPK-like)
26287	ANKRD2	ankyrin repeat domain 2 (stretch responsive muscle)

7321	UBE2D1	ubiquitin-conjugating enzyme E2D 1 (UBC4/5 homolog, yeast)
7323	UBE2D3	ubiquitin-conjugating enzyme E2D 3 (UBC4/5 homolog, yeast)
7323	UBE2D3	ubiquitin-conjugating enzyme E2D 3 (UBC4/5 homolog, yeast)
6662	SOX9	SRY (sex determining region Y)-box 9 (campomelic dysplasia, autosomal sex-reversal)
27238	GPKOW	G patch domain and KOW motifs
58472	SQRDL	sulfide quinone reductase-like (yeast)
26000	TBC1D10B	TBC1 domain family, member 10B
4218	RAB8A	RAB8A, member RAS oncogene family
26509	FER1L3	fer-1-like 3, myoferlin (C. elegans)
9079	LDB2	LIM domain binding 2
51187	C15orf15	chromosome 15 open reading frame 15
7922	SLC39A7	solute carrier family 39 (zinc transporter), member 7
1852	DUSP9	dual specificity phosphatase 9
56474	CTPS2	CTP synthase II
113	ADCY7	adenylate cyclase 7
6397	SEC14L1	SEC14-like 1 (S. cerevisiae)
1475	CSTA	cystatin A (stefin A)
55577	NAGK	N-acetylglucosamine kinase
8666	EIF3S4	eukaryotic translation initiation factor 3, subunit 4 delta, 44kDa
6574	SLC20A1	solute carrier family 20 (phosphate transporter), member 1
79053	ALG8	asparagine-linked glycosylation 8 homolog (S. cerevisiae, alpha-1,3-glucosyltransferase)
10483	SEC23B	Sec23 homolog B (S. cerevisiae)
4259	MGST3	microsomal glutathione S-transferase 3
5599	MAPK8	mitogen-activated protein kinase 8
10081	PDCD7	programmed cell death 7
5097	PCDH1	protocadherin 1 (cadherin-like 1)
64786	TBC1D15	TBC1 domain family, member 15
2060	EPS15	epidermal growth factor receptor pathway substrate 15
57708	MIER1	mesoderm induction early response 1 homolog (Xenopus laevis)
8452	CUL3	cullin 3
25957	C6orf111	chromosome 6 open reading frame 111
4817	NIT1	nitrilase 1
93621	MRFAP1	Mof4 family associated protein 1
51116	MRPS2	mitochondrial ribosomal protein S2
4915	NTRK2	neurotrophic tyrosine kinase, receptor, type 2
847	CAT	catalase
2982	GUCY1A3	guanylate cyclase 1, soluble, alpha 3
5997	RGS2	regulator of G-protein signalling 2, 24kDa

10133	OPTN	optineurin
64375	IKZF4	IKAROS family zinc finger 4 (Eos)
9246	UBE2L6	ubiquitin-conjugating enzyme E2L 6
23150	FRMD4B	FERM domain containing 4B
7257	TSNAX	translin-associated factor X
7187	TRAF3	TNF receptor-associated factor 3
9639	ARHGEF10	Rho guanine nucleotide exchange factor (GEF) 10
27069	GHITM	growth hormone inducible transmembrane protein
6286	S100P	S100 calcium binding protein P
83986	ITFG3	integrin alpha FG-GAP repeat containing 3
27229	76P	gamma tubulin ring complex protein (76p gene)
120863	DEPDC4	DEP domain containing 4
80790	CMIP	c-Maf-inducing protein
56852	RAD18	RAD18 homolog (S. cerevisiae)
26354	GNL3	guanine nucleotide binding protein-like 3 (nucleolar)
6146	RPL22	ribosomal protein L22
3909	LAMA3	laminin, alpha 3
7920	BAT5	HLA-B associated transcript 5
79573	TTC13	tetratricopeptide repeat domain 13
1843	DUSP1	dual specificity phosphatase 1
8829	NRP1	neuropilin 1
9518	GDF15	growth differentiation factor 15
10557	RPP38	ribonuclease P/MRP 38kDa subunit
9073	CLDN8	claudin 8
28512	NKIRAS1	NFKB inhibitor interacting Ras-like 1
8609	KLF7	Kruppel-like factor 7 (ubiquitous)
79084	WDR77	WD repeat domain 77
833	CARS	cysteinyl-tRNA synthetase
58487	ZF	HCF-binding transcription factor Zhangfei
2824	GPM6B	glycoprotein M6B
5264	PHYH	phytanoyl-CoA 2-hydroxylase
10418	SPON1	spondin 1, extracellular matrix protein
56882	CDC42SE1	CDC42 small effector 1
644	BLVRA	biliverdin reductase A
11221	DUSP10	dual specificity phosphatase 10
1731	SEPT1	septin 1
9424	KCNK6	potassium channel, subfamily K, member 6
5740	PTGIS	prostaglandin I2 (prostacyclin) synthase
7038	TG	thyroglobulin
827	CAPN6	calpain 6
3553	IL1B	interleukin 1, beta
150572	SMYD1	SET and MYND domain containing 1
23348	DOCK9	dedicator of cytokinesis 9
23288	IQCE	IQ motif containing E
60412	EXOC4	exocyst complex component 4
57680	CHD8	chromodomain helicase DNA binding protein 8

27248	C2orf30	chromosome 2 open reading frame 30
54987	C1orf123	chromosome 1 open reading frame 123
49855	ZNF291	zinc finger protein 291
22978	NT5C2	5'-nucleotidase, cytosolic II
26224	FBXL3	F-box and leucine-rich repeat protein 3
27243	CHMP2A	chromatin modifying protein 2A
5832	ALDH18A1	aldehyde dehydrogenase 18 family, member A1
2125	EVPL	envoplakin
6422	SFRP1	secreted frizzled-related protein 1
127544	IBRDC3	IBR domain containing 3
51703	ACSL5	acyl-CoA synthetase long-chain family member 5
85464	SSH2	slingshot homolog 2 (Drosophila)
114882	OSBPL8	oxysterol binding protein-like 8
55520	ELAC1	elaC homolog 1 (E. coli)
408	ARRB1	arrestin, beta 1
11178	LZTS1	leucine zipper, putative tumor suppressor 1
2762	GMDS	GDP-mannose 4,6-dehydratase
221322	C6orf170	chromosome 6 open reading frame 170
7175	TPR	translocated promoter region (to activated MET oncogene)
444	ASPH	aspartate beta-hydroxylase
84318	CCDC77	coiled-coil domain containing 77
471	ATIC	5-aminoimidazole-4-carboxamide ribonucleotide formyltransferase/IMP cyclohydrolase
966	CD59	CD59 molecule, complement regulatory protein
2828	GPR4	G protein-coupled receptor 4
57486	NLN	neurolysin (metallopeptidase M3 family)
1994	ELAVL1	ELAV (embryonic lethal, abnormal vision, Drosophila)-like 1 (Hu antigen R)
6484	ST3GAL4	ST3 beta-galactoside alpha-2,3-sialyltransferase 4
10902	BRD8	bromodomain containing 8
5664	PSEN2	presenilin 2 (Alzheimer disease 4)
5664	PSEN2	presenilin 2 (Alzheimer disease 4)
57685	CACHD1	cache domain containing 1
81567	TXNDC5	thioredoxin domain containing 5
4292	MLH1	mutL homolog 1, colon cancer, nonpolyposis type 2 (E. coli)
10252	SPRY1	sprouty homolog 1, antagonist of FGF signaling (Drosophila)
79817	MOBKL2B	MOB1, Mps One Binder kinase activator-like 2B (yeast)
9673	SLC25A44	solute carrier family 25, member 44
80144	FRAS1	Fraser syndrome 1
23469	PHF3	PHD finger protein 3
5002	SLC22A18	solute carrier family 22 (organic cation transporter), member 18

5204	PFDN5	prefoldin subunit 5
57216	VANGL2	vang-like 2 (van gogh, Drosophila)
9919	KIAA0310	KIAA0310
8809	IL18R1	interleukin 18 receptor 1
54454	KIAA1240	KIAA1240 protein
9904	RBM19	RNA binding motif protein 19
5522	PPP2R2C	protein phosphatase 2 (formerly 2A), regulatory subunit B (PR 52), gamma isoform
4664	NAB1	NGFI-A binding protein 1 (EGR1 binding protein 1)
27065	D4S234E	DNA segment on chromosome 4 (unique) 234 expressed sequence
51808	RNUXA	RNA U, small nuclear RNA export adaptor (phosphorylation regulated)
4045	LSAMP	limbic system-associated membrane protein
23515	MORC3	MORC family CW-type zinc finger 3
80824	DUSP16	dual specificity phosphatase 16
54881	TEX10	testis expressed sequence 10
10237	SLC35B1	solute carrier family 35, member B1
158399	ZNF483	zinc finger protein 483
9093	DNAJA3	DnaJ (Hsp40) homolog, subfamily A, member 3
10286	BCAS2	breast carcinoma amplified sequence 2
3678	ITGA5	integrin, alpha 5 (fibronectin receptor, alpha polypeptide)
64708	COPS7B	COP9 constitutive photomorphogenic homolog subunit 7B (Arabidopsis)
23301	EHBP1	EH domain binding protein 1
1910	EDNRB	endothelin receptor type B
57488	FAM62B	family with sequence similarity 62 (C2 domain containing) member B
55075	UACA	uveal autoantigen with coiled-coil domains and ankyrin repeats
6125	RPL5	ribosomal protein L5
221178	SPATA13	spermatogenesis associated 13
9669	EIF5B	eukaryotic translation initiation factor 5B
5054	SERPINE1	serpin peptidase inhibitor, clade E (nexin, plasminogen activator inhibitor type 1), member 1
1956	EGFR	epidermal growth factor receptor (erythroblastic leukemia viral (v-erb-b) oncogene homolog, avian)
55589	BMP2K	BMP2 inducible kinase
22837	COBLL1	COBL-like 1
6326	SCN2A2	sodium channel, voltage-gated, type II, alpha 2
8910	SGCE	sarcoglycan, epsilon
84900	TMEM118	transmembrane protein 118

10195	ALG3	asparagine-linked glycosylation 3 homolog (S. cerevisiae, alpha-1,3-mannosyltransferase)
27339	PRPF19	PRP19/PSO4 pre-mRNA processing factor 19 homolog (S. cerevisiae)
6671	SP4	Sp4 transcription factor
79656	C1orf165	chromosome 1 open reading frame 165
1192	CLIC1	chloride intracellular channel 1
5495	PPM1B	protein phosphatase 1B (formerly 2C), magnesium-dependent, beta isoform
29994	BAZ2B	bromodomain adjacent to zinc finger domain, 2B
65983	GRAMD3	GRAM domain containing 3
5864	RAB3A	RAB3A, member RAS oncogene family
2901	GRIK5	glutamate receptor, ionotropic, kainate 5
56995	TULP4	tubby like protein 4
8560	DEGS1	degenerative spermatocyte homolog 1, lipid desaturase (Drosophila)
25813	SAMM50	sorting and assembly machinery component 50 homolog (S. cerevisiae)
51097	SCCPDH	saccharopine dehydrogenase (putative)
116840	CNTROB	centrobin, centrosomal BRCA2 interacting protein
92454	PRR8	proline rich 8
60681	FKBP10	FK506 binding protein 10, 65 kDa
4199	ME1	malic enzyme 1, NADP(+)-dependent, cytosolic
4199	ME1	malic enzyme 1, NADP(+)-dependent, cytosolic
2113	ETS1	v-ets erythroblastosis virus E26 oncogene homolog 1 (avian)
51094	ADIPOR1	adiponectin receptor 1
404093	CUEDC1	CUE domain containing 1
2686	GGTL3	gamma-glutamyltransferase-like 3
128	ADH5	alcohol dehydrogenase 5 (class III), chi polypeptide
2145	EZH1	enhancer of zeste homolog 1 (Drosophila)
94241	TP53INP1	tumor protein p53 inducible nuclear protein 1
4839	NOL1	nucleolar protein 1, 120kDa
55758	RCOR3	REST corepressor 3
64223	GBL	G protein beta subunit-like
84826	SFT2D3	SFT2 domain containing 3
4072	TACSTD1	tumor-associated calcium signal transducer 1
11197	WIF1	WNT inhibitory factor 1
55743	CHFR	checkpoint with forkhead and ring finger domains
55701	FLJ10357	hypothetical protein FLJ10357
57134	MAN1C1	mannosidase, alpha, class 1C, member 1
152137	CCDC50	coiled-coil domain containing 50

1615	DARS	aspartyl-tRNA synthetase
7022	TFAP2C	transcription factor AP-2 gamma (activating enhancer binding protein 2 gamma)
92241	RCSD1	RCSD domain containing 1
10099	TSPAN3	tetraspanin 3
79086	C19orf42	chromosome 19 open reading frame 42
55149	PAPD1	PAP associated domain containing 1
9551	ATP5J2	ATP synthase, H+ transporting, mitochondrial F0 complex, subunit F2
2934	GSN	gelsolin (amyloidosis, Finnish type)
10806	SDCCAG8	serologically defined colon cancer antigen 8
1213	CLTC	clathrin, heavy chain (Hc)
1908	EDN3	endothelin 3
29015	SLC43A3	solute carrier family 43, member 3
6453	ITSN1	intersectin 1 (SH3 domain protein)
10516	FBLN5	fibulin 5
51340	CRNKL1	Crn, crooked neck-like 1 (Drosophila)
7586	ZKSCAN1	zinc finger with KRAB and SCAN domains 1
79441	C4orf15	chromosome 4 open reading frame 15
55643	BTBD2	BTB (POZ) domain containing 2
51704	GPRC5B	G protein-coupled receptor, family C, group 5, member B
201134	CCDC46	coiled-coil domain containing 46
10797	MTHFD2	methylenetetrahydrofolate dehydrogenase (NADP+ dependent) 2, methenyltetrahydrofolate cyclohydrolase
10914	PAPOLA	poly(A) polymerase alpha
3655	ITGA6	integrin, alpha 6
7517	XRCC3	X-ray repair complementing defective repair in Chinese hamster cells 3
1382	CRABP2	cellular retinoic acid binding protein 2
11218	DDX20	DEAD (Asp-Glu-Ala-Asp) box polypeptide 20
123263	MTFMT	mitochondrial methionyl-tRNA formyltransferase
6641	SNTB1	syntrophin, beta 1 (dystrophin-associated protein A1, 59kDa, basic component 1)
23385	NCSTN	nicastrin
55030	FBXO34	F-box protein 34
5360	PLTP	phospholipid transfer protein
10102	TSFM	Ts translation elongation factor, mitochondrial
7741	ZNF187	zinc finger protein 187
8503	PIK3R3	phosphoinositide-3-kinase, regulatory subunit 3 (p55, gamma)
142	PARP1	poly (ADP-ribose) polymerase family, member 1
50650	ARHGEF3	Rho guanine nucleotide exchange factor (GEF) 3

57414	RHBDD2	rhomboid domain containing 2
4660	PPP1R12B	protein phosphatase 1, regulatory (inhibitor) subunit 12B
3815	KIT	v-kit Hardy-Zuckerman 4 feline sarcoma viral oncogene homolog
29890	RBM15B	RNA binding motif protein 15B
1036	CDO1	cysteine dioxygenase, type I
91	ACVR1B	activin A receptor, type IB
2568	GABRP	gamma-aminobutyric acid (GABA) A receptor, pi
55627	SMPD4	sphingomyelin phosphodiesterase 4, neutral membrane (neutral sphingomyelinase-3)
92715	WDR85	WD repeat domain 85
79654	HECTD3	HECT domain containing 3
55697	VAC14	Vac14 homolog (S. cerevisiae)
10476	ATP5H	ATP synthase, H+ transporting, mitochondrial F0 complex, subunit d
51246	SCOTIN	scotin
4940	OAS3	2'-5'-oligoadenylate synthetase 3, 100kDa
55713	ZNF334	zinc finger protein 334
80023	NRSN2	neurensin 2
146198	ZFP90	zinc finger protein 90 homolog (mouse)
7169	TPM2	tropomyosin 2 (beta)
55276	PGM2	phosphoglucomutase 2
23623	RUSC1	RUN and SH3 domain containing 1
55501	CHST12	carbohydrate (chondroitin 4) sulfotransferase 12
143282	C10orf13	chromosome 10 open reading frame 13
23059	CLUAP1	clusterin associated protein 1
56990	CDC42SE2	CDC42 small effector 2
2041	EPHA1	EPH receptor A1
5430	POLR2A	polymerase (RNA) II (DNA directed) polypeptide A, 220kDa
9314	KLF4	Kruppel-like factor 4 (gut)
25831	HECTD1	HECT domain containing 1
266812	NAP1L5	nucleosome assembly protein 1-like 5
6944	VPS72	vacuolar protein sorting 72 (S. cerevisiae)
51167	CYB5R4	cytochrome b5 reductase 4
54622	ARL15	ADP-ribosylation factor-like 15
29083	GTPBP8	GTP-binding protein 8 (putative)
7043	TGFB3	transforming growth factor, beta 3
108	ADCY2	adenylate cyclase 2 (brain)
8939	FUBP3	far upstream element (FUSE) binding protein 3
10011	SRA1	steroid receptor RNA activator 1
5424	POLD1	polymerase (DNA directed), delta 1, catalytic subunit 125kDa
8694	DGAT1	diacylglycerol O-acyltransferase homolog 1 (mouse)
165215	KIAA1946	KIAA1946

2774	GNAL	guanine nucleotide binding protein (G protein), alpha activating activity polypeptide, olfactory type
7738	ZNF184	zinc finger protein 184
399687	MYO18A	myosin XVIIIA
85363	TRIM5	tripartite motif-containing 5
23008	KIAA0265	KIAA0265 protein
1837	DTNA	dystrobrevin, alpha
10600	USP16	ubiquitin specific peptidase 16
7570	ZNF22	zinc finger protein 22 (KOX 15)
7389	UROD	uroporphyrinogen decarboxylase
64116	SLC39A8	solute carrier family 39 (zinc transporter), member 8
81614	NIPA2	non imprinted in Prader-Willi/Angelman syndrome 2
11001	SLC27A2	solute carrier family 27 (fatty acid transporter), member 2
10298	PAK4	p21(CDKN1A)-activated kinase 4
6283	S100A12	S100 calcium binding protein A12
64419	C3orf29	chromosome 3 open reading frame 29
8559	PRPF18	PRP18 pre-mRNA processing factor 18 homolog (S. cerevisiae)
91050	DKFZp761B107	hypothetical protein DKFZp761B107
23203	PMPCA	peptidase (mitochondrial processing) alpha
84450	ZNF512	zinc finger protein 512
8031	NCOA4	nuclear receptor coactivator 4
29965	C16orf5	chromosome 16 open reading frame 5
23351	KIAA0323	KIAA0323
160760	PPTC7	PTC7 protein phosphatase homolog (S. cerevisiae)
3791	KDR	kinase insert domain receptor (a type III receptor tyrosine kinase)
51061	TXNDC11	thioredoxin domain containing 11
23225	NUP210	nucleoporin 210kDa
54894	RNF43	ring finger protein 43
9806	SPOCK2	sparc/osteonectin, cwcv and kazal-like domains proteoglycan (testican) 2
4640	MYO1A	myosin IA
8880	FUBP1	far upstream element (FUSE) binding protein 1
1456	CSNK1G3	casein kinase 1, gamma 3
317762	C14orf65	chromosome 14 open reading frame 65
22948	CCT5	chaperonin containing TCP1, subunit 5 (epsilon)
6853	SYN1	synapsin I
79017	C7orf24	chromosome 7 open reading frame 24
7402	UTRN	utrophin (homologous to dystrophin)
60559	SPCS3	signal peptidase complex subunit 3 homolog (S. cerevisiae)
7353	UFD1L	ubiquitin fusion degradation 1 like (yeast)

5066	PAM	peptidylglycine alpha-amidating monooxygenase
9925	ZBTB5	zinc finger and BTB domain containing 5
6596	SMARCA3	SWI/SNF related, matrix associated, actin dependent regulator of chromatin, subfamily a, member 3
51411	BIN2	bridging integrator 2
80325	ABTB1	ankyrin repeat and BTB (POZ) domain containing 1
813	CALU	calumenin
25875	LETMD1	LETM1 domain containing 1
54825	PCLKC	protocadherin LKC
54716	SLC6A20	solute carrier family 6 (proline IMINO transporter), member 20
91156	DKFZp434B1231	eEF1A2 binding protein
10539	TXNL2	thioredoxin-like 2
9933	KIAA0020	KIAA0020
1479	CSTF3	cleavage stimulation factor, 3' pre-RNA, subunit 3, 77kDa
23034	SAMD4A	sterile alpha motif domain containing 4A
84246	MED10	mediator of RNA polymerase II transcription, subunit 10 homolog (NUT2, S. cerevisiae)
3689	ITGB2	integrin, beta 2 (complement component 3 receptor 3 and 4 subunit)
65005	MRPL9	mitochondrial ribosomal protein L9
79758	DHRS12	dehydrogenase/reductase (SDR family) member 12
80271	ITPKC	inositol 1,4,5-trisphosphate 3-kinase C
246243	RNASEH1	ribonuclease H1
55315	SLC29A3	solute carrier family 29 (nucleoside transporters), member 3
56950	SMYD2	SET and MYND domain containing 2
6464	SHC1	SHC (Src homology 2 domain containing) transforming protein 1
10982	MAPRE2	microtubule-associated protein, RP/EB family, member 2
84173	RBED1	RNA binding motif and ELMO/CED-12 domain 1
25983	NGDN	neuroguidin, EIF4E binding protein
64333	ARHGAP9	Rho GTPase activating protein 9
51110	LACTB2	lactamase, beta 2
57605	PITPNM2	phosphatidylinositol transfer protein, membrane-associated 2
23316	CUTL2	cut-like 2 (Drosophila)
8987	GENX-3414	genethonin 1
11164	NUDT5	nudix (nucleoside diphosphate linked moiety X)-type motif 5
8541	PPFIA3	protein tyrosine phosphatase, receptor type, f polypeptide (PTPRF), interacting protein (liprin), alpha 3

10324	KBTBD10	kelch repeat and BTB (POZ) domain containing 10
7840	ALMS1	Alstrom syndrome 1
9712	USP6NL	USP6 N-terminal like
26156	RSL1D1	ribosomal L1 domain containing 1
25903	OLFML2B	olfactomedin-like 2B
6553	SLC9A5	solute carrier family 9 (sodium/hydrogen exchanger), member 5
9486	CHST10	carbohydrate sulfotransferase 10
10762	NUP50	nucleoporin 50kDa
63901	FAM111A	family with sequence similarity 111, member A
30827	CXXC1	CXXC finger 1 (PHD domain)
8463	TEAD2	TEA domain family member 2
5034	P4HB	procollagen-proline, 2-oxoglutarate 4-dioxygenase (proline 4-hydroxylase), beta polypeptide
6014	RIT2	Ras-like without CAAX 2
10464	C13orf24	chromosome 13 open reading frame 24
285704	RGMB	RGM domain family, member B
64376	IKZF5	IKAROS family zinc finger 5 (Pegasus)
85450	KIAA1754	KIAA1754
6738	TROVE2	TROVE domain family, member 2
25847	ANAPC13	anaphase promoting complex subunit 13
5074	PAWR	PRKC, apoptosis, WT1, regulator
9580	SOX13	SRY (sex determining region Y)-box 13
5810	RAD1	RAD1 homolog (S. pombe)
23061	TBC1D9B	TBC1 domain family, member 9B (with GRAM domain)
11164	NUDT5	nudix (nucleoside diphosphate linked moiety X)-type motif 5
123	ADFP	adipose differentiation-related protein
1827	DSCR1	Down syndrome critical region gene 1
6732	SRPK1	SFRS protein kinase 1
3219	HOXB9	homeobox B9
80209	C13orf23	chromosome 13 open reading frame 23
10745	PHTF1	putative homeodomain transcription factor 1
2992	GYG1	glycogenin 1
1880	EBI2	Epstein-Barr virus induced gene 2 (lymphocyte-specific G protein-coupled receptor)
26504	CNNM4	cyclin M4
57482	KIAA1211	KIAA1211 protein
64115	C10orf54	chromosome 10 open reading frame 54
64778	FNDC3B	fibronectin type III domain containing 3B
93624	MGC21874	transcriptional adaptor 2 (ADA2 homolog, yeast)-beta
203054	ADCK5	aarF domain containing kinase 5
23154	NCDN	neurochondrin
150465	TTL	tubulin tyrosine ligase
79767	ELMO3	engulfment and cell motility 3

57464	FAM40B	family with sequence similarity 40, member B
60625	DHX35	DEAH (Asp-Glu-Ala-His) box polypeptide 35
1386	ATF2	activating transcription factor 2
6813	STXBP2	syntaxin binding protein 2
6122	RPL3	ribosomal protein L3
8676	STX11	syntaxin 11
9404	LPXN	leupaxin
27352	RUTBC3	RUN and TBC1 domain containing 3
23277	KIAA0664	KIAA0664
55314	TMEM144	transmembrane protein 144
10714	POLD3	polymerase (DNA-directed), delta 3, accessory subunit
79009	DDX50	DEAD (Asp-Glu-Ala-Asp) box polypeptide 50
9725	TMEM63A	transmembrane protein 63A
50507	NOX4	NADPH oxidase 4
23122	CLASP2	cytoplasmic linker associated protein 2
80169	C17orf68	chromosome 17 open reading frame 68
23085	ERC1	ELKS/RAB6-interacting/CAST family member 1
58155	PTBP2	polypyrimidine tract binding protein 2
4140	MARK3	MAP/microtubule affinity-regulating kinase 3
220972	MARCH8	membrane-associated ring finger (C3HC4) 8
9899	SV2B	synaptic vesicle glycoprotein 2B
80700	UBXD1	UBX domain containing 1
4739	NEDD9	neural precursor cell expressed, developmentally down-regulated 9
4145	MATK	megakaryocyte-associated tyrosine kinase
26052	DNM3	dynamin 3
7559	ZNF12	zinc finger protein 12
4054	LTBP3	latent transforming growth factor beta binding protein 3
84254	CAMKK1	calcium/calmodulin-dependent protein kinase kinase 1, alpha
64342	HS1BP3	HCLS1 binding protein 3
55510	DDX43	DEAD (Asp-Glu-Ala-Asp) box polypeptide 43
10492	SYNCRIP	synaptotagmin binding, cytoplasmic RNA interacting protein
55677	IWS1	IWS1 homolog (S. cerevisiae)
6421	SFPQ	splicing factor proline/glutamine-rich (polypyrimidine tract binding protein associated)
53615	MBD3	methyl-CpG binding domain protein 3
9813	KIAA0494	KIAA0494
10494	STK25	serine/threonine kinase 25 (STE20 homolog, yeast)

10755	GIPC1	GIPC PDZ domain containing family, member 1
65010	SLC26A6	solute carrier family 26, member 6
199692	ZNF627	zinc finger protein 627
54809	SAMD9	sterile alpha motif domain containing 9
23552	CCRK	cell cycle related kinase
9063	PIAS2	protein inhibitor of activated STAT, 2
10131	TRAP1	TNF receptor-associated protein 1
50717	WDR42A	WD repeat domain 42A
9927	MFN2	mitofusin 2
9927	MFN2	mitofusin 2
8218	CLTCL1	clathrin, heavy chain-like 1
8218	CLTCL1	clathrin, heavy chain-like 1
6242	RTKN	rhotekin
10664	CTCF	CCCTC-binding factor (zinc finger protein)
57460	PPM1H	protein phosphatase 1H (PP2C domain containing)
50488	MINK1	misshapen-like kinase 1 (zebrafish)
23412	COMMD3	COMM domain containing 3
4171	MCM2	MCM2 minichromosome maintenance deficient 2, mitotin (S. cerevisiae)
23213	SULF1	sulfatase 1
5653	KLK6	kallikrein-related peptidase 6
55917	CTTNBP2NL	CTTNBP2 N-terminal like
55917	CTTNBP2NL	CTTNBP2 N-terminal like
9732	DOCK4	dedicator of cytokinesis 4
25938	C14orf125	chromosome 14 open reading frame 125
8895	CPNE3	copine III
10874	NMU	neuromedin U
114881	OSBPL7	oxysterol binding protein-like 7
64122	FN3K	fructosamine 3 kinase
84186	ZCCHC7	zinc finger, CCHC domain containing 7
1374	CPT1A	carnitine palmitoyltransferase 1A (liver)
27340	UTP20	UTP20, small subunit (SSU) processome component, homolog (yeast)
29964	C6orf49	chromosome 6 open reading frame 49
6821	SUOX	sulfite oxidase
112483	SAT2	spermidine/spermine N1-acetyltransferase 2
6434	SFRS10	splicing factor, arginine/serine-rich 10 (transformer 2 homolog, Drosophila)
4552	MTRR	5-methyltetrahydrofolate-homocysteine methyltransferase reductase
6809	STX3	syntaxin 3
4283	CXCL9	chemokine (C-X-C motif) ligand 9
57631	LRCH2	leucine-rich repeats and calponin homology (CH) domain containing 2
579	BAPX1	bagpipe homeobox homolog 1 (Drosophila)
23007	PLCH1	phospholipase C, eta 1
83892	KCTD10	potassium channel tetramerisation domain containing 10

283377	SPRYD4	SPRY domain containing 4
23246	BOP1	block of proliferation 1
109	ADCY3	adenylate cyclase 3
10989	IMMT	inner membrane protein, mitochondrial (mitofilin)
547	KIF1A	kinesin family member 1A
23463	ICMT	isoprenylcysteine carboxyl methyltransferase
79078	C1orf50	chromosome 1 open reading frame 50
26301	GBGT1	globoside alpha-1,3-N-acetylgalactosaminyltransferase 1
54617	INOC1	INO80 complex homolog 1 (S. cerevisiae)
6625	SNRP70	small nuclear ribonucleoprotein 70kDa polypeptide (RNP antigen)
7436	VLDLR	very low density lipoprotein receptor
3572	IL6ST	interleukin 6 signal transducer (gp130, oncostatin M receptor)
79876	UBE1DC1	ubiquitin-activating enzyme E1-domain containing 1
5257	PHKB	phosphorylase kinase, beta
10635	RAD51AP1	RAD51 associated protein 1
2926	GRSF1	G-rich RNA sequence binding factor 1
10979	PLEKHC1	pleckstrin homology domain containing, family C (with FERM domain) member 1
1501	CTNND2	catenin (cadherin-associated protein), delta 2 (neural plakophilin-related arm-repeat protein)
820	CAMP	cathelicidin antimicrobial peptide
221477	C6orf89	chromosome 6 open reading frame 89
26115	TANC2	tetratricopeptide repeat, ankyrin repeat and coiled-coil containing 2
55230	USP40	ubiquitin specific peptidase 40
23405	DICER1	Dicer1, Dcr-1 homolog (Drosophila)
1607	DGKB	diacylglycerol kinase, beta 90kDa
9685	CLINT1	clathrin interactor 1
3394	IRF8	interferon regulatory factor 8
857	CAV1	caveolin 1, caveolae protein, 22kDa
25948	KBTBD2	kelch repeat and BTB (POZ) domain containing 2
1153	CIRBP	cold inducible RNA binding protein
2219	FCN1	ficolin (collagen/fibrinogen domain containing) 1
55624	POMGNT1	protein O-linked mannose beta1,2-N-acetylglucosaminyltransferase
2057	EPOR	erythropoietin receptor
23435	TARDBP	TAR DNA binding protein
23435	TARDBP	TAR DNA binding protein
2213	FCGR2B	Fc fragment of IgG, low affinity IIb, receptor (CD32)
60686	C14orf93	chromosome 14 open reading frame 93

57553	MICAL3	microtubule associated monoxygenase, calponin and LIM domain containing 3
57510	XPO5	exportin 5
6405	SEMA3F	sema domain, immunoglobulin domain (Ig), short basic domain, secreted, (semaphorin) 3F
719	C3AR1	complement component 3a receptor 1
55118	CRTAC1	cartilage acidic protein 1
6608	SMO	smoothened homolog (Drosophila)
25999	CLIPR-59	CLIP-170-related protein
55224	ETNK2	ethanolamine kinase 2
400	ARL1	ADP-ribosylation factor-like 1
157247	MGC27345	hypothetical protein MGC27345
54838	C10orf26	chromosome 10 open reading frame 26
3667	IRS1	insulin receptor substrate 1
51760	SYT17	synaptotagmin XVII
9138	ARHGEF1	Rho guanine nucleotide exchange factor (GEF) 1
3658	IREB2	iron-responsive element binding protein 2
6035	RNASE1	ribonuclease, RNase A family, 1 (pancreatic)
284459	HKR1	GLI-Kruppel family member HKR1
115209	OMA1	OMA1 homolog, zinc metallopeptidase (S. cerevisiae)
5184	PEPD	peptidase D
55081	IFT57	intraflagellar transport 57 homolog (Chlamydomonas)
9658	ZNF516	zinc finger protein 516
1140	CHRNB1	cholinergic receptor, nicotinic, beta 1 (muscle)
2139	EYA2	eyes absent homolog 2 (Drosophila)
8318	CDC45L	CDC45 cell division cycle 45-like (S. cerevisiae)
8318	CDC45L	CDC45 cell division cycle 45-like (S. cerevisiae)
677	ZFP36L1	zinc finger protein 36, C3H type-like 1
6774	STAT3	signal transducer and activator of transcription 3 (acute-phase response factor)
84986	ARHGAP19	Rho GTPase activating protein 19
8027	STAM	signal transducing adaptor molecule (SH3 domain and ITAM motif) 1
4846	NOS3	nitric oxide synthase 3 (endothelial cell)
51093	C1orf66	chromosome 1 open reading frame 66
2214	FCGR3A	Fc fragment of IgG, low affinity IIIa, receptor (CD16a)
5033	P4HA1	procollagen-proline, 2-oxoglutarate 4-dioxygenase (proline 4-hydroxylase), alpha polypeptide I
51155	HN1	hematological and neurological expressed 1

146923	RUNDC1	RUN domain containing 1
57575	PCDH10	protocadherin 10
6171	RPL41	ribosomal protein L41
9840	KIAA0748	KIAA0748
5354	PLP1	proteolipid protein 1 (Pelizaeus-Merzbacher disease, spastic paraplegia 2, uncomplicated)
55719	C10orf6	chromosome 10 open reading frame 6
55614	C20orf23	chromosome 20 open reading frame 23
55612	C20orf42	chromosome 20 open reading frame 42
26035	GLCE	UDP-glucuronic acid epimerase
83543	C9orf58	chromosome 9 open reading frame 58
57326	PBXIP1	pre-B-cell leukemia transcription factor interacting protein 1
84059	GPR98	G protein-coupled receptor 98
10174	SORBS3	sorbin and SH3 domain containing 3
10174	SORBS3	sorbin and SH3 domain containing 3
27236	ARFIP1	ADP-ribosylation factor interacting protein 1 (arfaptin 1)
10607	TBL3	transducin (beta)-like 3
55727	BTBD7	BTB (POZ) domain containing 7
22881	ANKRD6	ankyrin repeat domain 6
79718	TBL1XR1	transducin (beta)-like 1X-linked receptor 1
714	C1QC	complement component 1, q subcomponent, C chain
761	CA3	carbonic anhydrase III, muscle specific
83719	YPEL3	yippee-like 3 (Drosophila)
1522	CTSZ	cathepsin Z
51373	MRPS17	mitochondrial ribosomal protein S17
4846	NOS3	nitric oxide synthase 3 (endothelial cell)
590	BCHE	butyrylcholinesterase
84498	FAM120B	family with sequence similarity 120B
9984	THOC1	THO complex 1
6498	SKIL	SKI-like
79939	SLC35E1	solute carrier family 35, member E1
10454	MAP3K7IP1	mitogen-activated protein kinase kinase kinase 7 interacting protein 1
10966	RAB40B	RAB40B, member RAS oncogene family
7113	TMPRSS2	transmembrane protease, serine 2
5920	RARRES3	retinoic acid receptor responder (tazarotene induced) 3
5523	PPP2R3A	protein phosphatase 2 (formerly 2A), regulatory subunit B'', alpha
55709	KBTBD4	kelch repeat and BTB (POZ) domain containing 4
9985	REC8L1	REC8-like 1 (yeast)
1300	COL10A1	collagen, type X, alpha 1(Schmid metaphyseal chondrodysplasia)
51136	LOC51136	PTD016 protein
58533	SNX6	sorting nexin 6

29997	GLTSCR2	glioma tumor suppressor candidate region gene 2
23586	DDX58	DEAD (Asp-Glu-Ala-Asp) box polypeptide 58
57701	KIAA1602	KIAA1602
30836	DNTTIP2	deoxynucleotidyltransferase, terminal, interacting protein 2
3291	HSD11B2	hydroxysteroid (11-beta) dehydrogenase 2
79188	TMEM43	transmembrane protein 43
3939	LDHA	lactate dehydrogenase A
2631	GBAS	glioblastoma amplified sequence
54940	OCIAD1	OCIA domain containing 1
9698	PUM1	pumilio homolog 1 (Drosophila)
4688	NCF2	neutrophil cytosolic factor 2 (65kDa, chronic granulomatous disease, autosomal 2)
2354	FOSB	FBJ murine osteosarcoma viral oncogene homolog B
27095	TRAPPC3	trafficking protein particle complex 3
57162	PELI1	pellino homolog 1 (Drosophila)
9727	RAB11FIP3	RAB11 family interacting protein 3 (class II)
79802	KIAA1822L	KIAA1822-like
7533	YWHAH	tyrosine 3-monooxygenase/tryptophan 5-monooxygenase activation protein, eta polypeptide
55215	KIAA1794	KIAA1794
29841	GRHL1	grainyhead-like 1 (Drosophila)
81563	C1orf21	chromosome 1 open reading frame 21
55858	TMEM165	transmembrane protein 165
79648	MCPH1	microcephaly, primary autosomal recessive 1
