# Supplementary material for: Evidence that molecular changes in cells occur before morphological alterations during the progression of breast ductal carcinoma
Source: Breast Cancer Res. 2008 Oct 17;10(5):R87. doi: 10.1186/bcr2157 (PMC2614523; doi:10.1186/bcr2157)
Supplement: Additional file 4 — Differentially expressed genes resulting from of 2 × 2 comparisons. Presented is a table listing the differentially expressed genes resulting from of 2 × 2 comparisons: non-neoplastic × pure DICS; non-neoplastic × in situ component of DCIS-IDC; and pure DCIS × in situ component of DCIS-IDC. [file bcr2157-S4.pdf]

Additional Data File 4 - Comparison two by two								
Non Neoplastic x pure DCIS			Non Neoplastic x DCIS-IDC			pure DCIS x DCIS-IDC		
GeneID	Symbol	Description	GeneID	Symbol	Description	GeneID	Symbol	Description
7402	UTRN	utrophin (homologous to dystrophin)	51061	TXNDC11	thioredoxin domain containing 11	64122	FN3K	fructosamine 3 kinase
9933	KIAA0020	KIAA0020	Inputs not found			4739	NEDD9	neural precursor cell expressed, developmentally down-regulated 9
Inputs not found			317762	C14orf65	chromosome 14 open reading frame 65	51061	TXNDC11	thioredoxin domain containing 11
Inputs not found			7402	UTRN	utrophin	29965	C16orf5	chromosome 16 open reading frame 5
51136	LOC51136	PTD016 protein	4140	MARK3	MAP/microtubule affinity-regulating kinase 3	51093	C1orf66	chromosome 1 open reading frame 66
317762	C14orf65	chromosome 14 open reading frame 65	29965	C16orf5	chromosome 16 open reading frame 5	23154	NCDN	neurochondrin
60686	C14orf93	chromosome 14 open reading frame 93	64122	FN3K	fructosamine 3 kinase	1140	CHRNB1	cholinergic receptor, nicotinic, beta 1 (muscle)
Inputs not found			23154	NCDN	neurochondrin	9486	CHST10	carbohydrate sulfotransferase 10
			114881	OSBPL7	oxysterol binding protein-like 7	Inputs not found		
			1140	CHRNB1	cholinergic receptor, nicotinic, beta 1 (muscle)	4140	MARK3	MAP/microtubule affinity-regulating kinase 3
			93624	MGC21874	transcriptional adaptor 2 (ADA2 homolog, yeast)-beta	114881	OSBPL7	oxysterol binding protein-like 7
			10539	TXNL2	thioredoxin-like 2	55917	CTTNBP2NL	CTTNBP2 N-terminal like
			Inputs not found			317762	C14orf65	chromosome 14 open reading frame 65
			55612	C20orf42	chromosome 20 open reading frame 42	93624	MGC21874	transcriptional adaptor 2 (ADA2 homolog, yeast)-beta
			23435	TARDBP	TAR DNA binding protein	Inputs not found		
			51093	C1orf66	chromosome 1 open reading frame 66	53615	MBD3	methyl-CpG binding domain protein 3

4739	NEDD9	neural precursor cell expressed, developmentally down-regulated 9	3394	IRF8	interferon regulatory factor 8
23034	SAMD4A	sterile alpha motif domain containing 4A	57605	PITPNM2	phosphatidylinositol transfer protein, membrane-associated 2
57605	PITPNM2	phosphatidylinositol transfer protein, membrane-associated 2	55612	C20orf42	chromosome 20 open reading frame 42
55917	CTTNBP2NL	CTTNBP2 N-terminal like	10539	TXNL2	thioredoxin-like 2
9580	SOX13	SRY (sex determining region Y)-box 13	23203	PMPCA	peptidase (mitochondrial processing) alpha
9486	CHST10	carbohydrate sulfotransferase 10	23034	SAMD4A	sterile alpha motif domain containing 4A
6122	RPL3	ribosomal protein L3	26301	GBGT1	globoside alpha-1,3-N-acetylgalactosaminyltransferase 1
54838	C10orf26	chromosome 10 open reading frame 26	54838	C10orf26	chromosome 10 open reading frame 26
2219	FCN1	ficolin (collagen/fibrinogen domain containing) 1	23435	TARDBP	TAR DNA binding protein
3394	IRF8	interferon regulatory factor 8	6122	RPL3	ribosomal protein L3
9933	KIAA0020	KIAA0020	Inputs not found		
53615	MBD3	methyl-CpG binding domain protein 3	23085	ERC1	ELKS/RAB6-interacting/CAST family member 1
Inputs not found			7840	ALMS1	Alstrom syndrome 1
23277	KIAA0664	KIAA0664	2219	FCN1	ficolin (collagen/fibrinogen domain containing) 1
23085	ERC1	ELKS/RAB6-interacting/CAST family member 1	5184	PEPD	peptidase D
54716	SLC6A20	solute carrier family 6 (proline IMINO transporter), member 20	7402	UTRN	utrophin

5184	PEPD	peptidase D	5920	RARRES3	retinoic acid receptor responder (tazarotene induced) 3
26301	GBGT1	globoside alpha-1,3-N-acetylgalactosaminyltransferase 1	80700	UBXD1	UBX domain containing 1
8676	STX11	syntaxin 11	55614	C20orf23	chromosome 20 open reading frame 23
Inputs not found			9580	SOX13	SRY (sex determining region Y)-box 13
55614	C20orf23	chromosome 20 open reading frame 23	Inputs not found		
3689	ITGB2	integrin, beta 2 (complement component 3 receptor 3 and 4 subunit)	54716	SLC6A20	solute carrier family 6 (proline IMINO transporter), member 20
9985	REC8	REC8 homolog (yeast)	23543	RBM9	RNA binding motif protein 9
5920	RARRES3	retinoic acid receptor responder (tazarotene induced) 3	Inputs not found		
719	C3AR1	complement component 3a receptor 1	10131	TRAP1	TNF receptor-associated protein 1
Inputs not found			23277	KIAA0664	KIAA0664
7840	ALMS1	Alstrom syndrome 1	3310	HSPA6	heat shock 70kDa protein 6 (HSP70B')
23543	RBM9	RNA binding motif protein 9	9985	REC8	REC8 homolog (yeast)
Inputs not found			55081	IFT57	intraflagellar transport 57 homolog (Chlamydomonas)
Inputs not found			Inputs not found		
6853	SYN1	synapsin I	3689	ITGB2	integrin, beta 2 (complement component 3 receptor 3 and 4 subunit)
23203	PMPCA	peptidase (mitochondrial processing) alpha	80271	ITPKC	inositol 1,4,5-trisphosphate 3-kinase C
Inputs not found			9404	LPXN	leupaxin
9404	LPXN	leupaxin	8676	STX11	syntaxin 11

51136	LOC51136	PTD016 protein	27340	UTP20	UTP20, small subunit (SSU) processome component, homolog (yeast)
60686	C14orf93	chromosome 14 open reading frame 93	Inputs not found		
55081	IFT57	intraflagellar transport 57 homolog (Chlamydomonas)	60625	DHX35	DEAH (Asp-Glu-Ala-His) box polypeptide 35
2057	EPOR	erythropoietin receptor	719	C3AR1	complement component 3a receptor 1
3310	HSPA6	heat shock 70kDa protein 6 (HSP70B')	57575	PCDH10	protocadherin 10
27236	ARFIP1	ADP-ribosylation factor interacting protein 1 (arfaptin 1)	7353	UFD1L	ubiquitin fusion degradation 1 like (yeast)
80271	ITPKC	inositol 1,4,5-trisphosphate 3-kinase C	5034	P4HB	procollagen-proline, 2-oxoglutarate 4-dioxygenase (proline 4-hydroxylase), beta polypeptide
Inputs not found			2057	EPOR	erythropoietin receptor
761	CA3	carbonic anhydrase III, muscle specific	6853	SYN1	synapsin I
2214	FCGR3A	Fc fragment of IgG, low affinity IIIa, receptor (CD16a)	Inputs not found		
6596	HLTF	helicase-like transcription factor	Inputs not found		
57575	PCDH10	protocadherin 10	Inputs not found		
1300	COL10A1	collagen, type X, alpha 1(Schmid metaphyseal chondrodysplasia)	160760	PPTC7	PTC7 protein phosphatase homolog (S. cerevisiae)
64333	ARHGAP9	Rho GTPase activating protein 9	64333	ARHGAP9	Rho GTPase activating protein 9

57464	FAM40B	family with sequence similarity 40, member B	8318	CDC45L	CDC45 cell division cycle 45-like (S. cerevisiae)
1386	ATF2	activating transcription factor 2	9933	KIAA0020	KIAA0020
27340	UTP20	UTP20, small subunit (SSU) processome component, homolog (yeast)	Inputs not found		
54825	PCLKC	protocadherin LKC	6596	HLTF	helicase-like transcription factor
6464	SHC1	SHC (Src homology 2 domain containing) transforming protein 1	820	CAMP	cathelicidin antimicrobial peptide
9685	CLINT1	clathrin interactor 1	9925	ZBTB5	zinc finger and BTB domain containing 5
9899	SV2B	synaptic vesicle glycoprotein 2B	123	ADFP	adipose differentiation-related protein
60625	DHX35	DEAH (Asp-Glu-Ala-His) box polypeptide 35	57464	FAM40B	family with sequence similarity 40, member B
84986	ARHGAP19	Rho GTPase activating protein 19	Inputs not found		
7353	UFD1L	ubiquitin fusion degradation 1 like (yeast)	54825	PCLKC	protocadherin LKC
10131	TRAP1	TNF receptor-associated protein 1	4054	LTBP3	latent transforming growth factor beta binding protein 3
10464	C13orf24	chromosome 13 open reading frame 24	10755	GIPC1	GIPC PDZ domain containing family, member 1
5523	PPP2R3A	protein phosphatase 2 (formerly 2A), regulatory subunit B'', alpha	547	KIF1A	kinesin family member 1A
590	BCHE	butyrylcholinesterase	2214	FCGR3A	Fc fragment of IgG, low affinity IIIa, receptor (CD16a)

57631	LRCH2	leucine-rich repeats and calponin homology (CH) domain containing 2			
8318	CDC45L	CDC45 cell division cycle 45-like (S. cerevisiae)	10982	MAPRE2	microtubule-associated protein, RP/EB family, member 2
Inputs not found			590	BCHE	butyrylcholinesterase
10762	NUP50	nucleoporin 50kDa	25983	NGDN	neuroguidin, EIF4E binding protein
54894	RNF43	ring finger protein 43	27236	ARFIP1	ADP-ribosylation factor interacting protein 1 (arfaptin 1)
51411	BIN2	bridging integrator 2	3791	KDR	kinase insert domain receptor (a type III receptor tyrosine kinase)
160760	PPTC7	PTC7 protein phosphatase homolog (S. cerevisiae)	6464	SHC1	SHC (Src homology 2 domain containing) transforming protein 1
820	CAMP	cathelicidin antimicrobial peptide	Inputs not found		
80700	UBXD1	UBX domain containing 1	1308	COL17A1	collagen, type XVII, alpha 1
25999	CLIP3	CAP-GLY domain containing linker protein 3	9685	CLINT1	clathrin interactor 1
3791	KDR	kinase insert domain receptor (a type III receptor tyrosine kinase)	10762	NUP50	nucleoporin 50kDa
9840	KIAA0748	KIAA0748	10464	C13orf24	chromosome 13 open reading frame 24
10324	KBTBD10	kelch repeat and BTB (POZ) domain containing 10	5523	PPP2R3A	protein phosphatase 2 (formerly 2A), regulatory subunit B'', alpha
26156	RSL1D1	ribosomal L1 domain containing 1	Inputs not found		
8218	CLTCL1	clathrin, heavy chain-like 1	79758	DHRS12	dehydrogenase/reductase (SDR family) member 12
Inputs not found			9063	PIAS2	protein inhibitor of activated STAT, 2

547	KIF1A	kinesin family member 1A	10324	KBTBD10	kelch repeat and BTB (POZ) domain containing 10
Inputs not found			677	ZFP36L1	zinc finger protein 36, C3H type-like 1
25847	ANAPC13	anaphase promoting complex subunit 13	9840	KIAA0748	KIAA0748
285704	RGMB	RGM domain family, member B	8895	CPNE3	copine III
9063	PIAS2	protein inhibitor of activated STAT, 2	57631	LRCH2	leucine-rich repeats and calponin homology (CH) domain containing 2
7113	TMPRSS2	transmembrane protease, serine 2	81566	FAM130A1	family with sequence similarity 130, member A1
84059	GPR98	G protein-coupled receptor 98	6553	SLC9A5	solute carrier family 9 (sodium/hydrogen exchanger), member 5
10979	PLEKHC1	pleckstrin homology domain containing, family C (with FERM domain) member 1	Inputs not found		
79758	DHRS12	dehydrogenase/reductase (SDR family) member 12	285704	RGMB	RGM domain family, member B
857	CAV1	caveolin 1, caveolae protein, 22kDa			
Inputs not found			55624	POMGNT1	protein O-linked mannose beta1,2-N-acetylglucosaminyltransferase
8987	GENX-3414	genethonin 1	9806	SPOCK2	sparc/osteonectin, cwcv and kazal-like domains proteoglycan (testican) 2
1522	CTSZ	cathepsin Z	54894	RNF43	ring finger protein 43
81566	FAM130A1	family with sequence similarity 130, member A1	55224	ETNK2	ethanolamine kinase 2

25983	NGDN	neuroguidin, EIF4E binding protein	51373	MRPS17	mitochondrial ribosomal protein S17
9925	ZBTB5	zinc finger and BTB domain containing 5	51136	LOC51136	PTD016 protein
4054	LTBP3	latent transforming growth factor beta binding protein 3	60686	C14orf93	chromosome 14 open reading frame 93
57226	LYRM2	LYR motif containing 2	1522	CTSZ	cathepsin Z
1880	EBI2	Epstein-Barr virus induced gene 2 (lymphocyte-specific G protein coupled receptor)	84059	GPR98	G protein-coupled receptor 98
123	ADFP	adipose differentiation-related protein	9899	SV2B	synaptic vesicle glycoprotein 2B
83892	KCTD10	potassium channel tetramerisation domain containing 10	84986	ARHGAP19	Rho GTPase activating protein 19
1153	CIRBP	cold inducible RNA binding protein	25847	ANAPC13	anaphase promoting complex subunit 13
1374	CPT1A	carnitine palmitoyltransferase 1A (liver)	761	CA3	carbonic anhydrase III, muscle specific
51373	MRPS17	mitochondrial ribosomal protein S17	23061	TBC1D9B	TBC1 domain family, member 9B (with GRAM domain)
10494	STK25	serine/threonine kinase 25 (STE20 homolog, yeast)	54617	INOC1	INO80 complex homolog 1 (S. cerevisiae)
Inputs not found			23246	BOP1	block of proliferation 1
			857	CAV1	caveolin 1, caveolae protein, 22kDa
23552	CCRK	cell cycle related kinase	51411	BIN2	bridging integrator 2
			2139	EYA2	eyes absent homolog 2 (Drosophila)
55224	ETNK2	ethanolamine kinase 2	1386	ATF2	activating transcription factor 2

10982	MAPRE2	microtubule-associated protein, RP/EB family, member 2	8218	CLTCL1	clathrin, heavy chain-like 1
Inputs not found			1374	CPT1A	carnitine palmitoyltransferase 1A (liver)
26504	CNNM4	cyclin M4	10979	PLEKHC1	pleckstrin homology domain containing, family C (with FERM domain) member 1
54617	INOC1	INO80 complex homolog 1 (S. cerevisiae)	579	BAPX1	bagpipe homeobox homolog 1 (Drosophila)
5034	P4HB	procollagen-proline, 2-oxoglutarate 4-dioxygenase (proline 4-hydroxylase), beta polypeptide	9927	MFN2	mitofusin 2
55230	USP40	ubiquitin specific peptidase 40	1153	CIRBP	cold inducible RNA binding protein
6813	STXBP2	syntaxin binding protein 2	57460	PPM1H	protein phosphatase 1H (PP2C domain containing)
83543	C9orf58	chromosome 9 open reading frame 58	57226	LYRM2	LYR motif containing 2
65010	SLC26A6	solute carrier family 26, member 6	26156	RSL1D1	ribosomal L1 domain containing 1
30827	CXXC1	CXXC finger 1 (PHD domain)	10494	STK25	serine/threonine kinase 25 (STE20 homolog, yeast)
5066	PAM	peptidylglycine alpha-amidating monooxygenase	Inputs not found		
Inputs not found			79017	C7orf24	chromosome 7 open reading frame 24
677	ZFP36L1	zinc finger protein 36, C3H type-like 1	6035	RNASE1	ribonuclease, RNase A family, 1 (pancreatic)
112483	SAT2	spermidine/spermine N1-acetyltransferase 2	84246	MED10	mediator of RNA polymerase II transcription, subunit 10 homolog (NUT2, S. cerevisiae)

57460	PPM1H	protein phosphatase 1H (PP2C domain containing)	6421	SFPQ	splicing factor proline/glutamine-rich (polypyrimidine tract binding protein associated)
54809	SAMD9	sterile alpha motif domain containing 9	Inputs not found		
57326	PBXIP1	pre-B-cell leukemia homeobox interacting protein 1	57482	KIAA1211	KIAA1211 protein
10755	GIPC1	GIPC PDZ domain containing family, member 1	51155	HN1	hematological and neurological expressed 1
1456	CSNK1G3	casein kinase 1, gamma 3	Inputs not found		
9806	SPOCK2	sparc/osteonectin, cwcv and kazal-like domains proteoglycan (testican) 2	6498	SKIL	SKI-like oncogene
714	C1QC	complement component 1, q subcomponent, C chain	8031	NCOA4	nuclear receptor coactivator 4
23246	BOP1	block of proliferation 1	50507	NOX4	NADPH oxidase 4
55624	POMGNT1	protein O-linked mannose beta1,2-N-acetylglucosaminyltransferase	22948	CCT5	chaperonin containing TCP1, subunit 5 (epsilon)
6553	SLC9A5	solute carrier family 9 (sodium/hydrogen exchanger), member 5	80209	C13orf23	chromosome 13 open reading frame 23
6608	SMO	smoothened homolog (Drosophila)	10745	PHTF1	putative homeodomain transcription factor 1
23061	TBC1D9B	TBC1 domain family, member 9B (with GRAM domain)	220972	8-Mar	membrane-associated ring finger (C3HC4) 8
64115	C10orf54	chromosome 10 open reading frame 54	9766	KIAA0247	KIAA0247

6014	RIT2	Ras-like without CAAX 2	1501	CTNND2	catenin (cadherin-associated protein), delta 2 (neural plakophilin-related arm-repeat protein)
10174	SORBS3	sorbin and SH3 domain containing 3	4283	CXCL9	chemokine (C-X-C motif) ligand 9
Inputs not found			146923	RUNDC1	RUN domain containing 1
Inputs not found			58155	PTBP2	polypyrimidine tract binding protein 2
9658	ZNF516	zinc finger protein 516			
80325	ABTB1	ankyrin repeat and BTB (POZ) domain containing 1	23603	CORO1C	coronin, actin binding protein, 1C
1607	DGKB	diacylglycerol kinase, beta 90kDa	23213	SULF1	sulfatase 1
5653	KLK6	kallikrein-related peptidase 6	10874	NMU	neuromedin U
79876	UBE1DC1	ubiquitin-activating enzyme E1-domain containing 1			
8895	CPNE3	copine III			
400	ARL1	ADP-ribosylation factor-like 1			
64376	IKZF5	IKAROS family zinc finger 5 (Pegasus)			
23351	KIAA0323	KIAA0323			
23316	CUTL2	cut-like 2 (Drosophila)			
2139	EYA2	eyes absent homolog 2 (Drosophila)			
2926	GRSF1	G-rich RNA sequence binding factor 1			
150465	TTL	tubulin tyrosine ligase			
8031	NCOA4	nuclear receptor coactivator 4			
1892	ECHS1	enoyl Coenzyme A hydratase, short chain, 1, mitochondrial			

23788	MTCH2	mitochondrial carrier homolog 2 (C. elegans)
84173	RBED1	RNA binding motif and ELMO/CED-12 domain 1
57482	KIAA1211	KIAA1211 protein
10745	PHTF1	putative homeodomain transcription factor 1
84246	MED10	mediator of RNA polymerase II transcription, subunit 10 homolog (NUT2, S. cerevisiae)
51422	PRKAG2	protein kinase, AMP-activated, gamma 2 non-catalytic subunit
80209	C13orf23	chromosome 13 open reading frame 23
79009	DDX50	DEAD (Asp-Glu-Ala-Asp) box polypeptide 50
6732	SRPK1	SFRS protein kinase 1
2992	GYG1	glycogenin 1
6821	SUOX	sulfite oxidase
8880	FUBP1	far upstream element (FUSE) binding protein 1
23413	FREQ	frequenin homolog (Drosophila)
55315	SLC29A3	solute carrier family 29 (nucleoside transporters), member 3
58155	PTBP2	polypyrimidine tract binding protein 2
1479	CSTF3	cleavage stimulation factor, 3' pre-RNA, subunit 3, 77kDa
246243	RNASEH1	ribonuclease H1

6809	STX3	syntaxin 3
23463	ICMT	isoprenylcysteine carboxyl methyltransferase
23412	COMMD3	COMM domain containing 3
50507	NOX4	NADPH oxidase 4
4283	CXCL9	chemokine (C-X-C motif) ligand 9
813	CALU	calumenin
60559	SPCS3	signal peptidase complex subunit 3 homolog (S. cerevisiae)
26115	TANC2	tetratricopeptide repeat, ankyrin repeat and coiled-coil containing 2
11164	NUDT5	nudix (nucleoside diphosphate linked moiety X)-type motif 5
23603	CORO1C	coronin, actin binding protein, 1C
51155	HN1	hematological and neurological expressed 1
220972	8-Mar	membrane-associated ring finger (C3HC4) 8
79017	C7orf24	chromosome 7 open reading frame 24
22948	CCT5	chaperonin containing TCP1, subunit 5 (epsilon)
23213	SULF1	sulfatase 1
146923	RUNDC1	RUN domain containing 1
6498	SKIL	SKI-like oncogene
9766	KIAA0247	KIAA0247
10874	NMU	neuromedin U
