# Supplementary material for: Evidence that molecular changes in cells occur before morphological alterations during the progression of breast ductal carcinoma
Source: Breast Cancer Res. 2008 Oct 17;10(5):R87. doi: 10.1186/bcr2157 (PMC2614523; doi:10.1186/bcr2157)
Supplement: Additional file 5 — 147 Genes differentially expressed between pure DCIS and in situ component of DCIS-IDC. Presented a table listing the 147 genes differentially expressed between pure DCIS and in situ component of DCIS-IDC with the respective fold changes. [file bcr2157-S5.pdf]

Additional Data File 5			
**Gene Symbol**	**Gene ID**	***In situ* component of DCIS-IDC x pure DCIS** (Fold Change)	**Functional Process**
ADFP	123	-2.79	unknown
ALMS1	7840	-3.8	visual perception, sensory perception of sound, response to stimulus
ANAPC13	25847	-2.19	cell division, ubiquitin cycle, cell cycle, mitosis,
ANKRD6	22881	-2.01	unknown
ARFIP1	27236	-2.55	regulation of protein secretion, intracellular protein transport, biological_process
ARHGAP19	84986	-2.2	signal transduction
ARHGAP9	64333	-2.92	signal transduction
ATF2	1386	-2.07	transcription, regulation of transcription, DNA-dependent,
BCHE	590	-2.62	cocaine metabolic process
BIN2	51411	-2.08	unknown
BOP1	23246	-2.1	rRNA processing
C10orf26	54838	-4.06	unknown
C13orf23	80209	2.41	unknown
C13orf24	10464	-2.49	unknown
C16orf5	29965	-5.45	apoptotic program, tumor necrosis factor-mediated signaling pathway, biological_process, induction of apoptosis, DNA damage response, signal transduction by p53 class mediator resulting in induction of apoptosis
C1orf66	51093	-5.32	unknown
C20orf23	55614	-3.6	microtubule-based movement, cell communication,
C20orf42	55612	-4.26	cell adhesion
C3AR1	719	-3.04	inflammatory response, G-protein coupled receptor protein signaling pathway, elevation of cytosolic calcium ion concentration, cell motility, signal transduction, chemotaxis, smooth muscle contraction, blood circulation, ,
C7orf24	79017	2.12	unknown
CA3	761	-2.18	one-carbon compound metabolic process
CAMP	820	-2.84	defense response to bacterium
CAV1	857	-2.08	cholesterol transport, vesicle organization and biogenesis, cholesterol homeostasis, regulation of blood coagulation,
CCT5	22948	2.4	protein folding
CDC45L	8318	-2.9	DNA replication initiation, DNA replication, cell cycle, DNA replication checkpoint,

CHRNB1	1140	-5.11	regulation of membrane potential, synaptic transmission, cholinergic, signal transduction, muscle contraction, cation transport
CHST10	9486	-5.07	carbohydrate metabolic process, cell adhesion,
CIRBP	1153	-2.04	response to cold
CLINT1	9685	-2.5	endocytosis
CLTCL1	8218	-2.07	protein complex assembly, anatomical structure morphogenesis, receptor-mediated endocytosis, intracellular protein transport, vesicle-mediated transport
COL17A1	1308	-2.52	cell-matrix adhesion, phosphate transport, epidermis development
CPNE3	8895	-2.38	lipid metabolic process, vesicle-mediated transport,
CPT1A	1374	-2.06	glucose metabolic process, lipid metabolic process, eating behavior, transport, regulation of insulin secretion, fatty acid beta-oxidation, fatty acid metabolic process
CTSZ	1522	-2.21	proteolysis
CTTNBP2NL	55917	-4.84	unknown
CXCL9	4283	2.7	inflammatory response, G-protein coupled receptor protein signaling pathway, immune response, signal transduction, chemotaxis, cell-cell signaling, cellular defense response
DHRS12	79758	-2.45	metabolic process
DHX35	60625	-3.07	RNA splicing, mRNA processing,
EPOR	2057	-2.99	elevation of cytosolic calcium ion concentration, heart development, brain development, signal transduction, small GTPase mediated signal transduction
ERC1	23085	-3.86	protein transport, positive regulation of anti-apoptosis, activation of NF-kappaB transcription factor, multicellular organismal development, I-kappaB phosphorylation, regulation of transcription, DNA-dependent
ETNK2	55224	-2.23	biological_process, mesodermal cell fate specification, metabolic process, multicellular organismal development, transcription, regulation of transcription, DNA-dependent
EYA2	2139	-2.08	multicellular organismal development
FAM40B	57464	-2.77	unknown
FCGR2B	2213	-3.39	immune response, signal transduction,
FCGR3A	2214	-2.7	immune response
FCN1	2219	-3.79	opsonization, phosphate transport, signal transduction
FN3K	64122	-6.3	fructoselysine metabolic process
GBGT1	26301	-4.06	glycolipid biosynthetic process, carbohydrate metabolic process,

GIPC1	10755	-2.74	G-protein coupled receptor protein signaling pathway
GPR98	84059	-2.21	neuropeptide signaling pathway, photoreceptor cell maintenance, cell-cell adhesion, sensory perception of sound, signal transduction, neurological system process, nervous system development, maintenance of organ identity, ,
HN1	51155	2.3	unknown
IFT57	55081	-3.3	regulation of apoptosis, caspase activation, transcription, regulation of transcription, DNA-dependent,
IMMT	10989	-2.92	biological_process
INOC1	54617	-2.11	chromatin remodeling
IRF8	3394	-4.33	immune response, myeloid cell differentiation, negative regulation of transcription from RNA polymerase II promoter, transcription, regulation of transcription, DNA-dependent
ITGB2	3689	-3.27	neutrophil chemotaxis, inflammatory response, apoptosis, cell-matrix adhesion, leukocyte adhesion, cell-cell signaling, cell adhesion, regulation of peptidyl-tyrosine phosphorylation, ,
ITPKC	80271	-3.26	unknown
KBTBD10	10324	-2.42	ubiquitin cycle, striated muscle contraction,
KDR	3791	-2.55	transmembrane receptor protein tyrosine kinase signaling pathway, protein amino acid phosphorylation, cell differentiation, angiogenesis, multicellular organismal development
KIAA0664	23277	-3.45	translation
KIAA0748	9840	-2.38	unknown
KIAA1211	57482	2.3	unknown
KIF1A	547	-2.71	anterograde axon cargo transport, microtubule-based movement,
LETMD1	25875	-2.32	unknown
LPXN	9404	-3.2	protein complex assembly, signal transduction, cell adhesion
LRCH2	57631	-2.33	unknown
LTBP3	4054	-2.74	unknown
MAPRE2	10982	-2.62	cell division, signal transduction, cell cycle, cell proliferation, cellular defense response, mitosis
MARCH8	220972	2.45	immune response, ubiquitin cycle,
MARK3	4140	-4.91	protein amino acid phosphorylation
MBD3	53615	-4.58	transcription, regulation of transcription, DNA-dependent,
MED10	84246	2.15	transcription, regulation of transcription, DNA-dependent,
MGC21874	93624	-4.7	transcription, regulation of transcription, DNA-dependent,
MRPS17	51373	-2.23	transport, translation,
NCDN	23154	-5.19	unknown

NCOA4	8031	2.34	androgen receptor signaling pathway, transmembrane receptor protein tyrosine kinase signaling pathway, positive regulation of transcription, DNA-dependent, male gonad development, transcription
NEDD9	4739	-6.02	cell division, regulation of cell growth, actin filament bundle formation, signal transduction, cell cycle, cell adhesion, integrin-mediated signaling pathway, cytoskeleton organization and biogenesis, ,
NGDN	25983	-2.59	rRNA processing, regulation of translation,
NMU	10874	4.12	NMU, neuropeptide signaling pathway, regulation of smooth muscle contraction, digestion, signal transduction,
NOX4	50507	2.35	inflammatory response, cell morphogenesis, cell aging, oxygen and reactive oxygen species metabolic process, superoxide release, negative regulation of cell proliferation
NUP50	10762	-2.5	protein transport, intracellular transport, mRNA transport, intracellular protein transport across a membrane,
OSBPL7	114881	-4.91	steroid metabolic process, lipid transport,
P4HA1	5033	2.2	protein metabolic process
P4HB	5034	-3.01	peptidyl-proline hydroxylation to 4-hydroxy-L-proline, cell redox homeostasis,
PCDH10	57575	-3.01	homophilic cell adhesion, cell adhesion,
PCLKC	54825	-2.75	homophilic cell adhesion, cell adhesion, negative regulation of cell growth
PEPD	5184	-3.65	collagen catabolic process, proteolysis, amino acid metabolic process
PHTF1	10745	2.42	transcription, regulation of transcription, DNA-dependent,
PIAS2	9063	-2.43	androgen receptor signaling pathway, ubiquitin cycle, positive regulation of transcription, DNA-dependent, transcription,
PITPNM2	57605	-4.28	metabolic process, transport,
PLEKHC1	10979	-2.05	actin cytoskeleton organization and biogenesis, cell adhesion, regulation of cell shape
PMPCA	23203	-4.2	proteolysis
POLD3	10714	-2.69	mismatch repair, DNA synthesis during DNA repair,
POMGNT1	55624	-2.25	protein amino acid O-linked glycosylation, protein amino acid N-linked glycosylation,

PPM1H	57460	-2.02	unknown
PPP2R3A	5523	-2.48	protein amino acid dephosphorylation
PPTC7	160760	-2.92	unknown
PTBP2	58155	2.84	RNA splicing, mRNA processing,
RAD51AP1	10635	2.96	DNA repair, double-strand break repair via homologous recombination,
RARRES3	5920	-3.64	negative regulation of cell proliferation
REC8L1	ND	-3.37	meiotic recombination, meiosis, spermatogenesis, sister chromatid cohesion,
RGMB	285704	-2.28	positive regulation of transcription, signal transduction, BMP signaling pathway, cell adhesion,
RNF43	54894	-2.23	unknown
RPL3	6122	-4.02	translation
RPL41	6171	-3.52	translation
RSL1D1	26156	-2	translation
RUNDC1	146923	2.76	unknown
SAMD4A	23034	-4.11	positive regulation of translation
SHC1	6464	-2.54	positive regulation of cell proliferation, regulation of cell growth, regulation of epidermal growth factor receptor activity, intracellular signaling cascade, epidermal growth factor receptor signaling pathway, activation of MAPK activity, positive regulation of mitosis
SKIL	6498	2.34	signal transduction, transcription from RNA polymerase II promoter, multicellular organismal development
SLC6A20	54716	-3.54	neurotransmitter transport
SLC9A5	6553	-2.32	sodium ion transport, ion transport, regulation of pH
SMARCA3	ND	-2.84	regulation of transcription from RNA polymerase II promoter, chromatin modification, transcription
SOX13	9580	-3.58	anatomical structure morphogenesis, regulation of transcription, DNA-dependent,
SPOCK2	9806	-2.24	extracellular matrix organization and biogenesis, synaptogenesis, regulation of cell differentiation
STK25	10494	-2	signal transduction, response to oxidative stress, protein amino acid phosphorylation
STX11	8676	-3.18	intracellular protein transport, membrane fusion, vesicle-mediated transport
SULF1	23213	3.92	apoptosis, metabolic process, heparan sulfate proteoglycan metabolic process
SV2B	9899	-2.21	neurotransmitter transport
SYN1	6853	-2.97	synaptic transmission, neurotransmitter secretion,

TARDBP	23435	-4.03	RNA splicing, transcription from RNA polymerase II promoter, mRNA processing, transcription, mitosis, regulation of transcription, DNA-dependent
TBC1D9B	23061	-2.14	regulation of Rab GTPase activity
TRAP1	10131	-3.5	biological_process, protein folding,
TXNDC11	51061	-6	cell redox homeostasis
TXNL2	10539	-4.24	cell redox homeostasis
UBXD1	80700	-3.63	biological_process
UFD1L	7353	-3.01	skeletal development, ubiquitin cycle, ubiquitin-dependent protein catabolic process
UTP20	27340	-3.16	negative regulation of cell proliferation
ZBTB5	9925	-2.82	transcription, regulation of transcription, DNA-dependent,
ZFP36L1	677	-2.4	mRNA catabolic process, deadenylation-dependent decay, regulation of mRNA stability, vasculogenesis, regulation of translation,
RABEPK	10244	-4.94	receptor-mediated endocytosis, vesicle docking during exocytosis,
RHOU	58480	-3.12	small GTPase mediated signal transduction
GPR77	27202	-2.52	G-protein coupled receptor protein signaling pathway, signal transduction, chemotaxis, biological_process,
KCTD15	79047	-2.35	potassium ion transport
AOF2	23028	-2.46	transcription, regulation of transcription, DNA-dependent,
LSM4	25804	-2.85	RNA splicing, mRNA processing,
IGSF6	10261	2.32	immune response, cell surface receptor linked signal transduction,
CORO1C	23603	3.05	signal transduction, phagocytosis,
AZGP1	563	-2.94	immune response, antigen processing and presentation, cell adhesion
DENND3	22898	-3.51	unknown
DGCR2	9993	-2.96	cell adhesion, organ morphogenesis,
KPNA6	23633	-2.32	NLS-bearing substrate import into nucleus, intracellular protein transport,
B4GALT5	9334	-2.77	carbohydrate metabolic process
LOX	4015	2.01	transmembrane receptor protein tyrosine kinase signaling pathway, collagen fibril organization, protein modification process, elastic fiber assembly, blood vessel development, lung development
VPS54	51542	-2.25	protein transport, retrograde transport, endosome to Golgi,
FGFBP1	9982	-3.28	signal transduction, negative regulation of cell proliferation, cell-cell signaling

GOSR2	9570	-3.97	ER to Golgi vesicle-mediated transport, intracellular protein transport, membrane fusion
